# Functional Specialization of the Plant miR396 Regulatory Network through Distinct MicroRNA–Target Interactions

**DOI:** 10.1371/journal.pgen.1002419

**Published:** 2012-01-05

**Authors:** Juan M. Debernardi, Ramiro E. Rodriguez, Martin A. Mecchia, Javier F. Palatnik

**Affiliations:** Instituto de Biología Molecular y Celular de Rosario (IBR), Consejo Nacional de Investigaciones Científicas y Técnicas (CONICET) and Facultad de Ciencias Bioquímicas y Farmacéuticas, Universidad Nacional de Rosario, Rosario, Argentina; Penn State, United States of America

## Abstract

MicroRNAs (miRNAs) are ∼21 nt small RNAs that regulate gene expression in animals and plants. They can be grouped into families comprising different genes encoding similar or identical mature miRNAs. Several miRNA families are deeply conserved in plant lineages and regulate key aspects of plant development, hormone signaling, and stress response. The ancient miRNA miR396 regulates conserved targets belonging to the *GROWTH-REGULATING FACTOR* (*GRF*) family of transcription factors, which are known to control cell proliferation in *Arabidopsis* leaves. In this work, we characterized the regulation of an additional target for miR396, the transcription factor *bHLH74*, that is necessary for *Arabidopsis* normal development. *bHLH74* homologs with a miR396 target site could only be detected in the sister families *Brassicaceae* and *Cleomaceae.* Still, *bHLH74* repression by miR396 is required for margin and vein pattern formation of *Arabidopsis* leaves. MiR396 contributes to the spatio-temporal regulation of *GRF* and *bHLH74* expression during leaf development. Furthermore, a survey of miR396 sequences in different species showed variations in the 5′ portion of the miRNA, a region known to be important for miRNA activity. Analysis of different miR396 variants in *Arabidopsis thaliana* revealed that they have an enhanced activity toward *GRF* transcription factors. The interaction between the *GRF* target site and miR396 has a bulge between positions 7 and 8 of the miRNA. Our data indicate that such bulge modulates the strength of the miR396-mediated repression and that this modulation is essential to shape the precise spatio-temporal pattern of *GRF2* expression. The results show that ancient miRNAs can regulate conserved targets with varied efficiency in different species, and we further propose that they could acquire new targets whose control might also be biologically relevant.

## Author Summary

Plants and other multicellular organisms need precise spatio-temporal control of gene expression, and this regulatory capacity depends, in part, on small RNAs. MicroRNAs (miRNAs) are one class of ∼21 nt small RNAs that originate from endogenous fold-back precursors found in plants and animals. They recognize complementary target sites in target mRNAs and guide them to cleavage or translational arrest. Studies of conserved miRNA networks in *Arabidopsis* and other plants have revealed that they fulfill essential regulatory roles. Most of the ancient miRNAs regulate transcription factors involved in plant development and hormone signaling. Here, we characterize the miR396 regulatory network. While miR396 regulates *GRF* transcription factors, at least in angiosperms and gymnosperms, this miRNA additionally regulates another transcription factor of the bHLH class but only in *Arabidopsis thaliana* and closely related species. Most conspicuously, the regulation of both conserved and new targets is important for leaf development in *Arabidopsis*. We also show that miRNA variants can exist in certain species and that they can display an enhanced activity towards their targets. In summary, we propose that conserved miRNA regulatory networks might expand their functions by the recruitment of additional targets as well as by slight variations in the small RNA sequences.

## Introduction

MicroRNAs (miRNAs) are small RNA molecules, ∼21 nt in length, that are widespread regulators of gene expression in animals and plants [reviewed in [1], [2]]. They recognize target RNAs by base complementarity and guide them to cleavage or translational arrest [1]. MiRNA-encoding genes are transcribed as primary transcripts harboring a fold-back structure with the miRNA embedded in one of its arms. In plants, these precursors are processed in the nucleus by the ribonuclease III DICER-LIKE1 (DCL1) together with accessory components [reviewed in [3]]. The outcome of this processing activity is a miRNA/miRNA* duplex, which is 2′-O-methylated by HEN1 and incorporated into an AGO complex [reviewed in [1]].

The current version of the miRNA database (miRBase 17.0) states the existence of more than 200 *MIRNAs* in *Arabidopsis thaliana* [4]. In many cases, *MIRNA*-genes can be grouped into families comprising different *loci* encoding similar or identical mature miRNAs [5]. However, the majority of the miRNAs in *Arabidopsis* are single young molecules indicating that their generation is a frequent process [6], [7], [8], [9], [10]. It is unclear whether these recently evolved miRNAs have a relevant biological contribution [11], [12]. One exception might be the regulation of *AGL16* by the young miR824, which participates in stomatal development in *Arabidopsis* [13]. On the other hand, twenty-one miRNAs families are conserved in angiosperms, with some of them even present in lycopods and bryophytes [9], [11], [14]. In contrast to the younger ones, conserved miRNAs regulate key aspects of plant development, hormone signaling and stress response [11].

While plant miRNAs have extensive base-pairing to their targets, the interaction along the 5′ portion of the miRNA is the most relevant feature for its activity [15], [16]. Interestingly, variations in the sequence of the small RNA among family members can have consequences on miRNA specificity [17]. In *Arabidopsis*, the miR159/miR319 family of miRNAs comprises six small RNAs that share 17 out of their 21 nt and regulate transcription factors of the *TCP* and *MYB* classes [16], [17], [18], [19], [20]. While miR319 can guide both types of targets to cleavage [17], miR159 can only affect the *MYBs* [16], [17], [19].

MiRNA miR396 regulates *GROWTH-REGULATING FACTORs* (*GRFs*) [21], [22], [23], [24], a plant specific family of transcription factors known to be involved in the control of cell proliferation during leaf development [22], [24], [25], [26], [27], [28]. The interaction between miR396 and the *GRFs* is unusual in plants as it contains a bulge in the 5′ region [22], [23]. MiR396 accumulates with leaf age and restricts the pattern of expression of the *GRFs* to the proliferative region of the organ [22]. Overexpression of the miRNA causes a drastic reduction in cell number, while abolishing the regulation of *GRF2* by miR396 promotes a moderate increase in organ size [22]. Interestingly, variations in the miR396 sequence can be found in different species (miRBase 17.0), such as a base insertion in the 5′ region of the miRNA, which is found only in rice and other monocotyledons [29], [30].

It has been observed since the discovery of plant miRNAs that ancient miRNAs usually recognize similarly conserved target genes [16], [23], [31], [32]. The occurrence of non-conserved targets recently incorporated during evolution to pre-existing miRNA networks has not been systematically studied so far, and more importantly, it is unknown whether the regulation of newly acquired targets has biological significance. Furthermore, miRNAs can have variations in their mature sequences in different species, which could potentially lead to neo-functionalization of the small RNA regulatory networks. Here, we characterize the miR396 regulatory network. We demonstrate its expansion to regulate a new target in species related to *Arabidopsis thaliana* and provide evidence pointing to the importance of this regulation in *Arabidopsis* development. We also show that monocot-specific variants of miR396 display an enhanced activity towards the conserved *GRF* transcription factors, while the sub-optimal regulation of the *GRFs* by miR396 in *Arabidopsis* might be important to quantitatively control *GRF* levels.

## Results

### Analysis of miR396 targets

**Figure 1:**
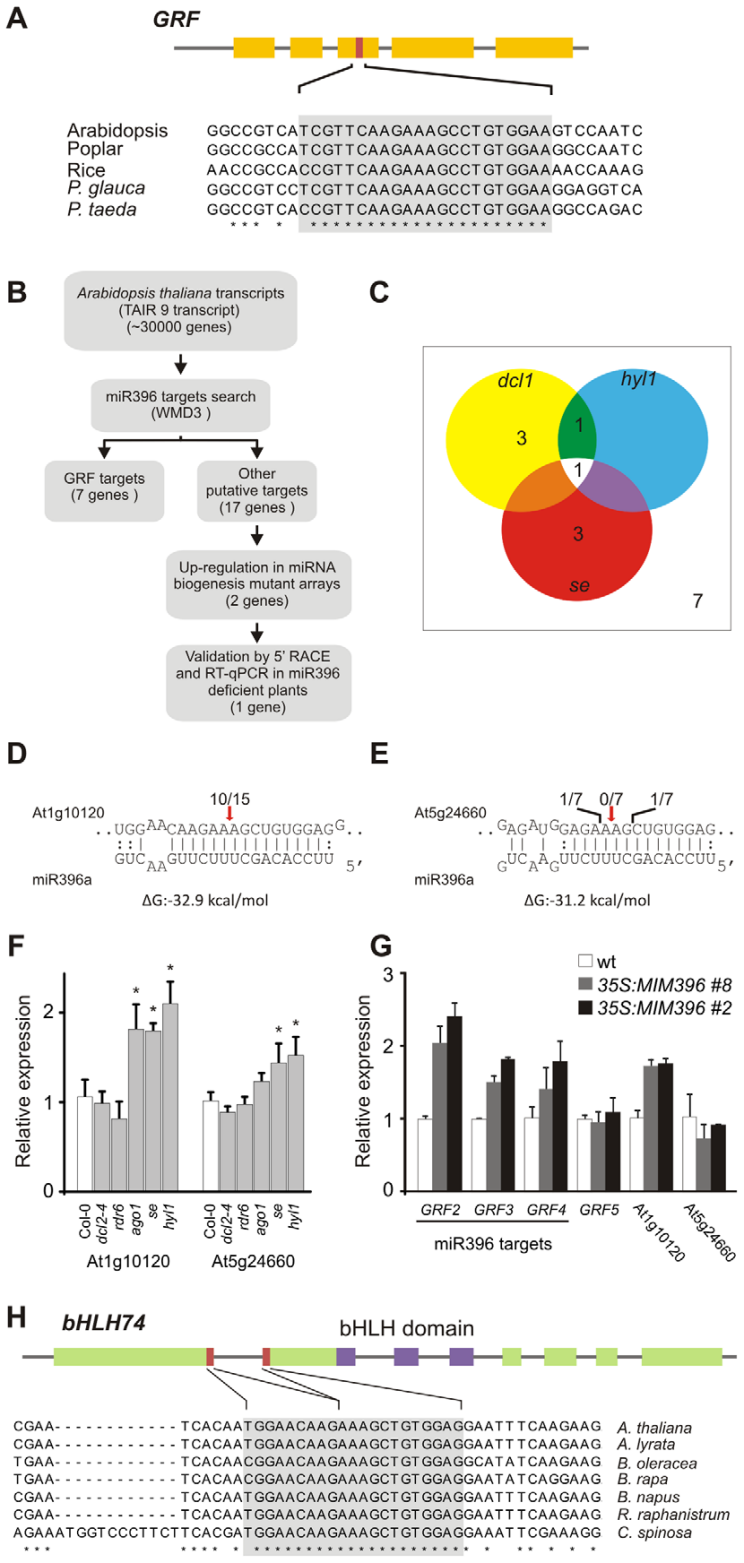
Analysis of potential miR396 targets in *Arabidopsis thaliana*. (A) Scheme representing a typical *GRF* gene and the conservation of the target site in selected angiosperm and gymnosperm species. Conserved positions across all species are indicated by asterisks. Note that the number of exons might vary among *GRF* genes. See Table S4 for accession numbers of sequences used. (B) Scheme representing the strategy used to identify new miR396 targets of potential biological significance. Target search was performed over the TAIR9 database using the WMD3 target search tool (http://wmd3.weigelworld.org/), allowing 5 mismatches and gaps in the miRNA-target pairs. Predicted targets are shown in Table S1. (C) Diagram showing putative miR396 targets that are up-regulated at least 30% in miRNA mutants [32], [35]. Expression levels were obtained from Genevestigator (www.genevestigator.com). (D,E) Modified RACE-PCR mapping of At1g10120 and At5g24660 mRNA cleavage sites. Red arrows indicate predicted miR396 cleavage sites. (F) At1g10120 and At5g24660 transcript levels in different small RNA mutant plants estimated by RT-qPCR. Data shown are mean ± SEM of 3 biological replicates. See Table S7 for a list of mutant alleles used. (G) At1g10120 and At5g24660 transcript levels in plants expressing an artificial target-mimic against miR396 (*MIM396*) estimated by RT-qPCR. Data shown are mean ± SEM of 3 biological replicates. (H) Scheme representing the At1g10120 locus. The miR396 target site is formed after the splicing of the first two exons. Target site conservation in several species is indicated (see Table S4 for accession numbers of sequences used). Conserved positions across all species are indicated by asterisks.

*GRF* regulation by miR396 is conserved at least in angiosperms and gymnosperms based on the presence of the small RNA [14], [33] and *GRF* transcription factors harboring the miR396 target site (Figure 1A). We began analyzing the existence of additional miR396 targets by searching the rice, poplar and *Arabidopsis* genomes using empirically-derived miRNA-target rules [16]. Transcription factors of the *GRF* class are the only conserved targets among these species (see Tables S1, S2, S3) [23], and their regulation by miR396 is known to be relevant for *Arabidopsis* development [22].

We also observed that 17, 26 and 12 additional potential target genes that do not encode *GRF* transcription factors are predicted in *Arabidopsis*, poplar and rice, respectively (see Tables S1, S2, S3). Detection of RNAs cleaved at positions 10–11 with respect to cognate small RNAs is a hallmark of miRNA activity [34], however, these products do not necessarily indicate a biologically relevant process. Therefore, we studied additional and potential miR396 targets from several points of view to identify those targets whose regulation would be more likely to have biological importance (Figure 1B). Similar integrated strategies have previously allowed the identification of ta-siRNA targets [32].

First, we analyzed the expression of the 17 predicted targets in mutants of the miRNA biogenesis pathway in *Arabidopsis* using published ATH1 microarray data [32], [35]. We found that two genes, At5g24660 and At1g10120 were up-regulated at least 30% in the miRNA biogenesis mutants *hyl1*, *serrate* (*se*) and *dcl1* (Figure 1C; see Table S1). This increase was similar to that observed for the miR396-regulated *GRFs* (see Table S1). At1g10120 analysis by a modified 5′ RACE-PCR revealed mRNA fragments consistent with a miR396-guided cleavage (Figure 1D) in agreement with previous results obtained for this gene by genome-wide analysis of miRNA targets [36], [37]. In contrast, we did not find any pattern of miR396 activity on At5g24660 (Figure 1E).

We then performed a RT-qPCR with oligos spanning miR396 cognate sites in several siRNA and miRNA biogenesis mutants (Figure 1F). At1g10120 was up-regulated two-fold in *ago1*, *se* and *hyl1* mutants, while no change was detected in *dcl2-4* or *rdr6* mutants (Figure 1F). At5g24660 showed a moderate increase only in *se* and *hyl1* mutants (Figure 1F), albeit to a lower extent than that observed for At1g10120.

Finally, we prepared transgenic plants expressing an artificial target mimic directed against miR396 (*MIM396*) to decrease the endogenous miRNA activity. These plants did not have any obvious phenotypic defects, similar to a previously described *MIM396* prepared along a collection of target-mimics [12], probably due to remaining miR396 activity. However, at the molecular level, we found that At1g10120 was up-regulated two-fold in *35S:MIM396* plants, which was analogous to the increase observed in miR396-regulated *GRFs* (Figure 1G). In contrast, transcript levels of At5g24660 and *GRF5*, which is not regulated by miR396, were unaffected by the expression of *MIM396* (Figure 1G). Taken together, these results indicate that miR396 has a meaningful impact on the RNA regulation of this new target and that this regulation is quantitatively similar to the one observed in conserved *GRFs*.

At1g10120 encodes a basic Helix-Loop-Helix (bHLH) transcription factor, namely bHLH74 [38], [39], [40]. Interestingly, we observed that the miR396-binding site of *bHLH74* is formed after the splicing of the first two exons (Figure 1H). We searched for *bHLH74* homologs with a miR396 target site in EST and genome sequence databases of species related to *Arabidopsis thaliana*. Our observations indicated that the miR396-*bHLH74* regulatory module is present in *Brassicaceae* species. We also found that conservation extends to *Cleome spinosa* which belongs to the sister family *Cleomaceae*, separated 40–50 million years from *Arabidopsis thaliana* [41], [42] (Figure 1H; see Figure S1 and Table S4).

We did not find evidence of *bHLH74* homologs with a miR396 target site in more distant species of *Arabidopsis thaliana*, either looking at syntenic regions of sequenced genomes such as poplar or by BLAST search against EST databases. The search was also performed trying to identify relaxed target sites, for example, looking only at the 5′ region which is completely located in the second exon of *bHLH74* without intron interruption. In neither case did we find an additional *bHLH74* homologue that could be potentially regulated by miR396.

### Regulation of *bHLH74* by miR396

**Figure 2:**
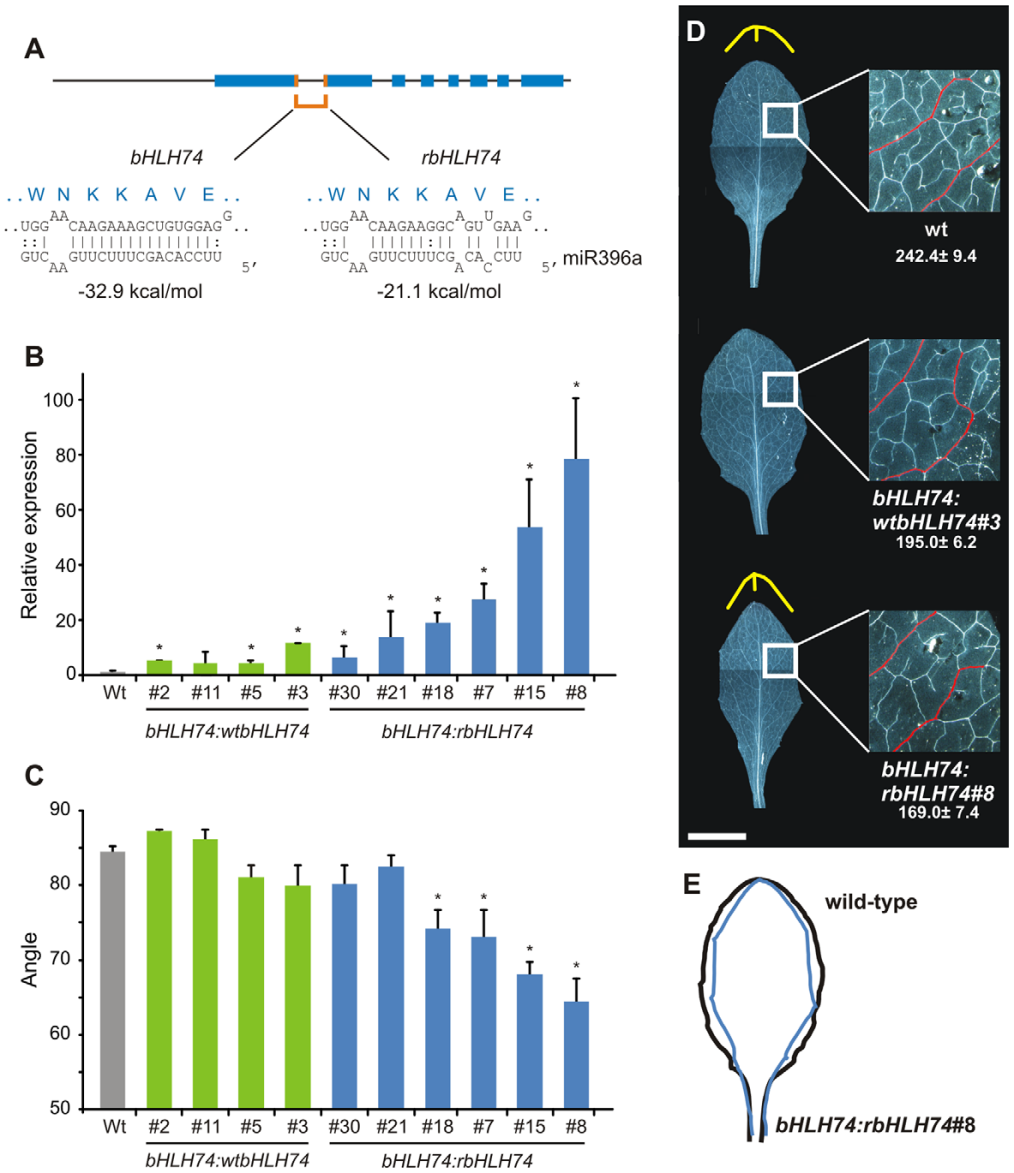
Characterization of transgenic plants expressing a miR396-resistant *bHLH74*. (A) Schematic representation of *bHLH74* and *rbHLH74* constructs. (B) *bHLH74* transcript levels estimated by RT-qPCR in mature leaves of plants expressing a wild-type (*bHLH74:wtbHLH74*) or miR396-resistant (*bHLH74:rbHLH74*) form of the gene encoding the transcription factor. Data shown are mean ± SEM of 3 biological replicates. Asterisks indicate significant differences between transgenic and wild-type plants, as determined by ANOVA (P<0.05). (C) Angle determination at the distal tip of leaf #5 in transgenics depicted on panel (B). Asterisks indicate significant differences between transgenics and wild-type plants, as determined by ANOVA (P<0.05). (D) Morphology of fully-expanded leaf #5. The different angles at the distal edge of the leaf are highlighted in yellow for wild-type and *bHLH74:rbHLH74* leaves. An inset on the right shows the difference in venation (secondary veins are highlighted in red), with the number of branching points (NBP) indicated below. Data shown are mean ± SEM of 8 biological replicates. Scale Bar: 1 cm. (E) Scheme highlighting differences in leaf edges of wild-type and miR396-resistant *bHLH74* plants.

The up-regulation of *bHLH74* in miR396-deficient plants and its conservation in a group of related species suggested that miR396 regulation might have a biological significance. To study the importance of *bHLH74* regulation by miR396 in more detail, we prepared a version of the gene with mutations that impaired its interaction with the miRNA (*rbHLH74*). We introduced silent mutations to avoid changing the encoded amino acids and did not modify the region next to the intron-exon junction (Figure 2A).

First, we prepared transgenic plants expressing the wild-type and miR396 resistant gene from the viral CaMV 35S promoter. We observed that both transgenes were able to cause developmental defects, however the effects caused by the overexpression of the miRNA resistant gene (*35S:rbHLH74*) were stronger and in the most extreme cases led to the formation of chlorotic seedlings, which failed to develop shoot apical meristems (see Figure S2). These results demonstrate that high levels of *bHLH74* can be toxic for normal plant development and that miR396 can down-regulate *bHLH74* expression levels *in vivo*.

Next, two vectors expressing a genomic version of the transcription factor with different sensitivities to miR396, including the endogenous upstream regulatory regions, were constructed. For an initial characterization of the resulting transgenic plants, we analyzed *bHLH74* transcript levels in mature leaves. As miR396 accumulates with leaf age [22], we expected large differences in *bHLH74* mRNA abundance in these samples. The genomic version of the transcription factor containing silent mutations that impaired its regulation by miR396 (*bHLH74*:*rbHLH74*) accumulated varied levels of mRNA reaching levels eighty-fold higher than those for the endogenous transcript in the most extreme cases (Figure 2B). On the other hand, the wild-type version (*bHLH74:wtbHLH74*) accumulated at most eight-fold more (Figure 2B). These results are consistent with high levels of endogenous miR396 guiding the cleavage of the wild-type *bHLH74* transcript. Note that the differences in mRNA accumulation between *bHLH74*:*rbHLH74* and *bHLH74*:*bHLH74* are smaller in younger developing tissues, where miR396 levels are low (see below).

These transgenic plants also displayed alterations in leaf development, especially in the vein pattern and organ shape, which had sharper edges than those of wild-type leaves (Figure 2D–2E). The angle formed at the distal part of the leaf was significantly reduced in most *bHLH74:rbHLH74* transgenics (Figure 2C–2D). Furthermore, there was also a reduction in the number of branching points (NBP) in the vasculature [43] in plants with high *bHLH74* levels (Figure 2D, inset). These results show that *bHLH74* regulation by miR396 might be biologically important for *Arabidopsis* development.

### A *bhlh74* mutant has leaf developmental changes opposite to *rbHLH74*

**Figure 3:**
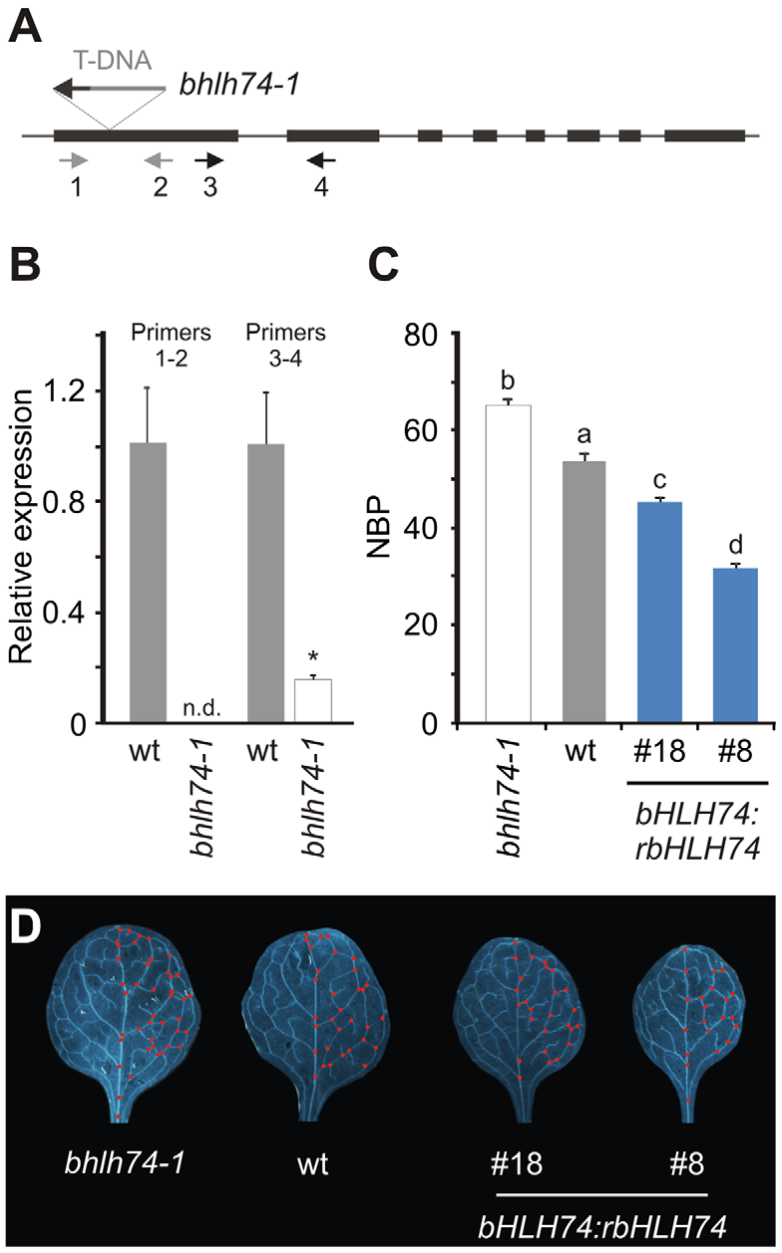
Analysis of *bhlh74* mutants. (A) Scheme of the *bHLH74* locus showing the T-DNA insertion corresponding to the GABI-Kat 720G11 line. Arrows indicate the pairs of primers (1–2 or 3–4, see also Table S7) used to quantify *bHLH74* transcript levels by RT-qPCR. (B) *bHLH74* transcript levels in wt and *bhlh74-1* (GABI-Kat 720G11) seedlings (12–day old), using pairs of primers shown in (A). Data shown are mean ± SEM of 3 biological replicates; n.d.: not detected. Asterisks indicate significant differences between mutant and wild-type plants, as determined by ANOVA (P<0.05). (C) Number of branching points (NBP) in fully-expanded first leaves of wt, *bhlh74-1* and *bHLH74:rbHLH74* (lines #18 and #8) plants. Bars marked with different letters are significantly different as determined by ANOVA and Duncan's multiple range test (P<0.05). (D) Fully-expanded leaves (#1) from wt, *bhlh74-1* and *bHLH74:rbHLH74* (lines #18 and #8) plants. Red dots highlight branching points.

The analysis of transgenic plants harboring a *rbHLH74* transgene suggested that *bHLH74* might play a role during *Arabidopsis* leaf development. To further explore this possibility, we identified a loss-of-function mutant for transcription factor *bhlh74-1* (Figure 3A). Determination of *bHLH74* mRNA levels indicated that they were severely reduced in this mutant (Figure 3B).

As *rbHLH74* caused a reduction in the number of branching points in the leaf vasculature, we analyzed NBP values for *bhlh74* mutants. Interestingly, we found that the NBP was increased approximately 24% in *bhlh74-1* with respect to the wild type (Figure 3C–3D). This phenotype was opposite to that found in plants harboring a *rbHLH74* transgene. Actually, we observed a quantitative response between the increase of *rbHLH74* mRNA levels and the reduction of NBP (Figure 2B and Figure 3C–3D). Together with previous reports [22], these results show that both types of miR396 targets, namely *GRFs* and *bHLH74*, have biological roles during leaf development.

### MiR396 contributes to the spatio-temporal expression of *bHLH74* and *GRF2*

**Figure 4:**
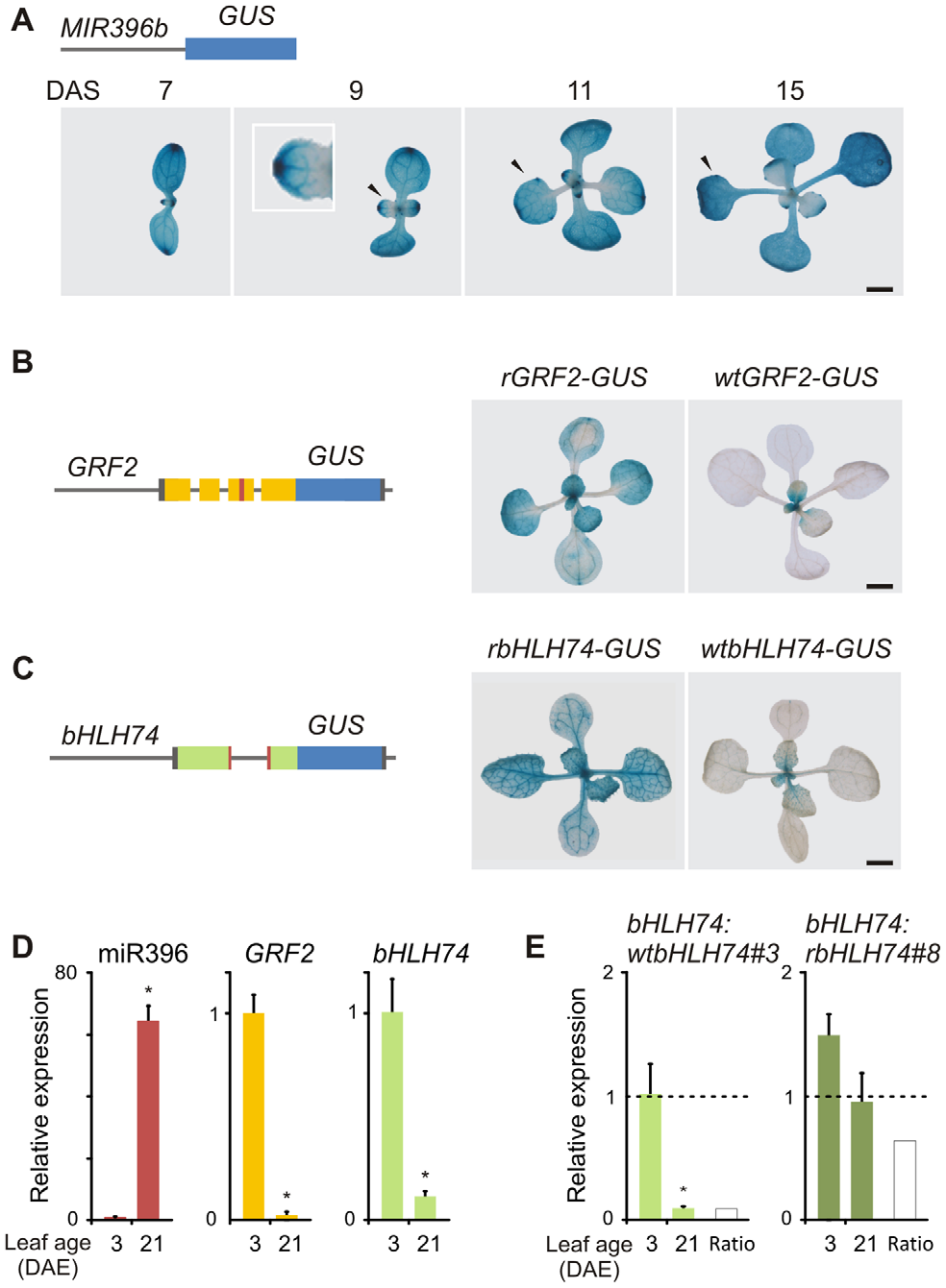
MiR396 coordinates the spatio-temporal expression of *bHLH74* and *GRF2*. (A) GUS staining of *MIR396b:GUS* plants of different age. Arrowheads show leaf #1. Numerals indicate plant age expressed in DAS (Days After Sowing). The inset shows a closer look at a developing leaf displaying a miR396 expression gradient. (B) GUS staining of *wtGRF2-GUS* (right) and *rGRF2-GUS* lines (15-day old). *rGRF2-GUS* has synonymous mutations in the miR396-target site. Left, scheme representing the reporters. The upstream regulatory regions and the first 4 exons were fused to GUS. The miRNA-target site is indicated in red. (C) GUS staining of *wtbHLH74-GUS* (right) and *rbHLH74-GUS* lines (15-day old). *rbHLH74-GUS* has synonymous mutations in the miR396 target site. Left, scheme representing the reporters. The upstream regulatory regions and the first 2 exons were fused to GUS. The miRNA target site is indicated in red. (D) miR396, *GRF2* and *bHLH74* RNA levels estimated by RT-qPCR in wild-type leaf #5 (DAE: Days After leaf #5 Emergence). Data shown are mean ± SEM of 3 biological replicates. Asterisks indicate significant differences between leaves, as determined by ANOVA (P<0.05). (E) *bHLH74* expression levels at different ages of leaf #5 in *bHLH74:wtbHLH74* and *bHLH74:rbHLH74* plants. Expression levels were normalized to 3 DAE of the *bHLH74:wtbHLH74* line #*3*. Data are mean ± SEM of 3 biological replicates. Asterisks indicate significant differences between leaves, as determined by ANOVA (P<0.05). Scale Bars: 2 mm.

We then compared the regulation of the new *bHLH74* target by miR396 to the regulation of an ancient *GRF* target, such as *GRF2*. First, we prepared a reporter to follow miR396 expression by fusing the 2 Kb upstream regulatory sequences of *MIR396b* to a GUS reporter. The miRNA reporter increased its expression during leaf development (Figure 4A). In young leaves *MIR396b:GUS* was expressed in a gradient along the longitudinal axis of the leaf, with higher expression at the distal part (Figure 4A). At later developing stages, *MIR396b:GUS* was detected in whole organs (Figure 4A). The profile of the reporter matched previous small RNA blots performed for this miRNA [22].

A wild-type reporter for *GRF2* containing the upstream regulatory region as well as the first four exons harboring the miR396 target site was expressed in young leaves and in proximo-distal gradient along the longitudinal axis of the organ (Figure 4B) [22]. Interestingly, the pattern of *MIR396b* and *GRF2* expression was complementary during leaf development. This was especially noticeable in 15-day old seedlings where *MIR396b* was expressed in older organs and in the distal part of young leaves, while *GRF2* was expressed in the proximal part of young organs (Figure 4A–4B). A miR396-resistant *GRF2* reporter, with mutations in the target site, was expressed in a broader domain, highlighting the activity of the miRNA in shaping *GRF2* expression (Figure 4B) [22].

We then prepared two reporters to follow the regulation of *bHLH74* by miR396. We fused its promoter and the first two exons, which generate the miRNA target site, to the *GUS* gene (Figure 4C). This first sensor, which has a functional miR396-binding site (*wtbHLH74-GUS*) was strongly expressed in young leaves, especially in the veins, while its expression decreased in older leaves (10 out of 16 independent transgenic plants) (Figure 4C). The second sensor contained silent mutations in the miRNA binding site, which impaired its regulation by miR396 (*rbHLH74-GUS*). This reporter was expressed in organs much older than those of the wild-type version highlighting the role of miR396 in restricting its activity to younger organs (12 out of 16 independent transgenic plants) (Figure 4C). Although we cannot disregard the possibility of the existence of other regulatory levels affecting *bHLH74* expression such as post-translational modifications, which are not detected by our sensors, the results show that miR396 contributes to the spatio-temporal regulation of *bHLH74.* Furthermore, the expression of the *bHLH74* sensors in the veins is in agreement with the biological role of the transcription factor in the control of leaf vasculature development.

We then analyzed miR396, *GRF2* and *bHLH74* transcript levels by RT-qPCR in young and fully-expanded leaves (Figure 4D). We observed that while miR396 was induced several times during leaf development, both *GRF2* and *bHLH74* decreased significantly. These quantitative measurements are in accordance with the whole-mount GUS stainings, supporting the function of miR396 in the regulation of both types of targets during organ growth (Figure 4A–4D).

Finally, we measured the accumulation of *bHLH74* mRNA in transgenic plants expressing the wild-type and miR396 resistant version of *bHLH74* at two leaf developmental stages. At young stages when miR396 levels are low, *bHLH74* was only slightly higher in *bHLH74:rbHLH74* than in *bHLH74:wtbHLH74* transgenic plants (Figure 4E). However, the wild-type *bHLH74* was significantly down-regulated more than ten times in older organs, in agreement with the activation of miR396 expression (Figure 4E). These results further support the role of miR396 in the regulation of *bHLH74* expression.

### Variations among miR396 family members in plants

The fact that *bHLH74* regulation by miR396 is important for *Arabidopsis* development suggests that the miR396 regulatory network could be relatively dynamic at least with respect to the acquisition of new targets. We then explored whether there could be biologically relevant variations in the miRNA.

**Figure 5:**
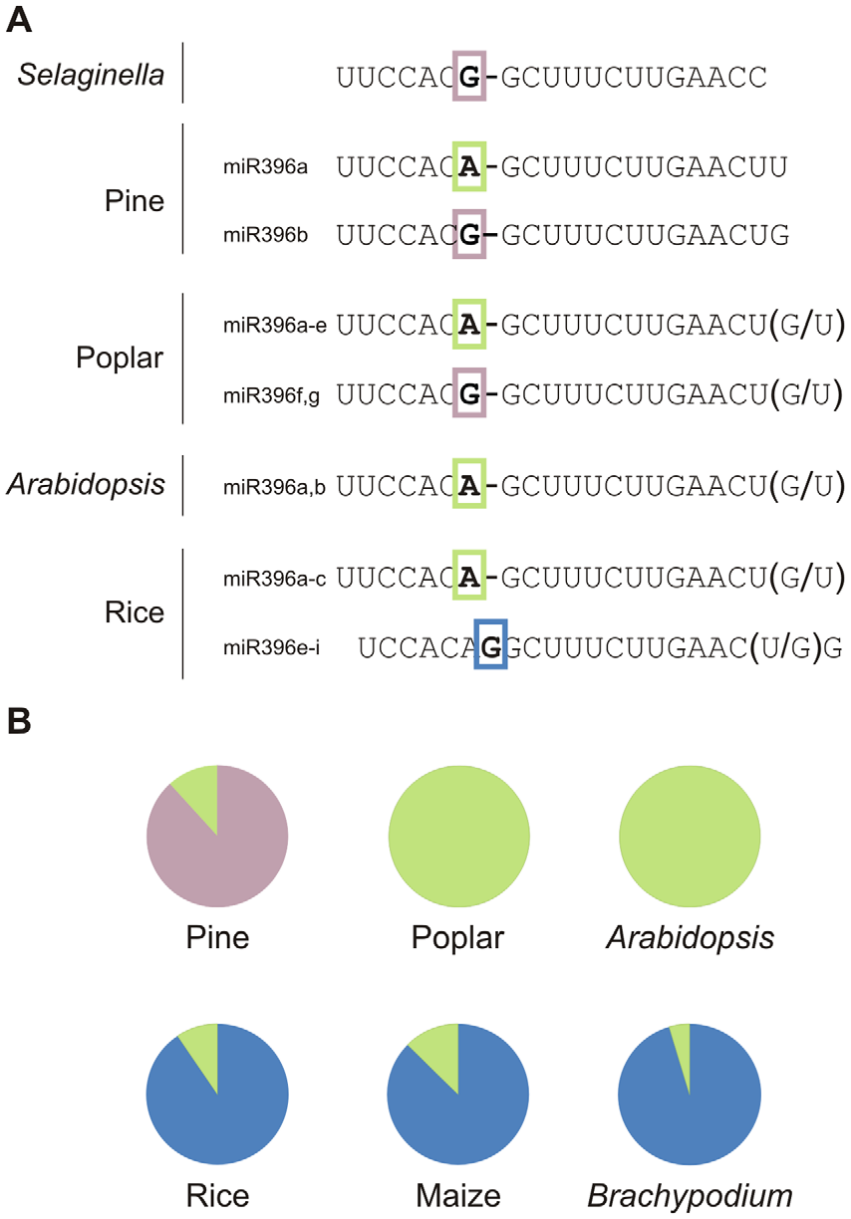
Variations among miR396 family members in plants. (A) MiR396 family composition of selected species. Differences in the 5′ region are highlighted with colored boxes, while variations at the 3′ end for each case are indicated in parentheses. (B) Diagrams showing the relative abundance of miR396 variants in pine, poplar, Arabidopsis and monocot libraries according to deep sequencing data (see Table S5).

Analysis of miR396 variants in different species using the miRNA database (miRBase 17.0) indicates that there are, indeed, several variants of miR396 (Figure 5). In *Selaginella*, miR396 has a G at position 7, while both genes in *Arabidopsis* encode small RNAs with an A at that position (Figure 5A). Pine and poplar have precursors for both types of mature miR396 species (Figure 5A). Interestingly, there is a miR396 variant with an insertion of a G at position 7–8 of the miRNA (Figure 5A). This variant was first detected in rice [29], and further studies indicated that it is found only in monocotyledons (Figure 5B) [29], [30], [44].

Sequence differences among related miRNAs could be important in plants as it has been shown for miR319 and miR159, which have very similar sequences but regulate different genes [17]. Interestingly, the changes observed for the miR396 sequence at position 7–8 are predicted to have an important effect on miRNA activity based on previous biochemical data and mutant analysis [15], [16], [17]. We also searched for potential variations in other ancient miRNAs annotated in the miRNA database (miRBase 17.0), and found that changes in the 5′ region of the miRNA might also exist in other cases (see Figure S3).

It should be considered that some of the polymorphisms observed could arise from non-expressed paralogs that have been annotated due to their homology to other known miRNAs. To confirm the expression of the miR396 sequences, we analyzed publicly available deep-sequencing small RNA libraries from several species [29], [33], [45], [46], [47], [48], [49], [50]. We found that most small RNAs were detected *in vivo*, confirming that a complex spectrum of miR396 sequences co-exists in nature (Figure 5B; see Table S5). Interestingly, we observed that the monocot-specific variant was the most abundant miR396 variant in rice, maize and *Brachypodium distachyon* as judged by deep-sequencing (Figure 5B).

### Differential activity of miR396 variants

**Figure 6:**
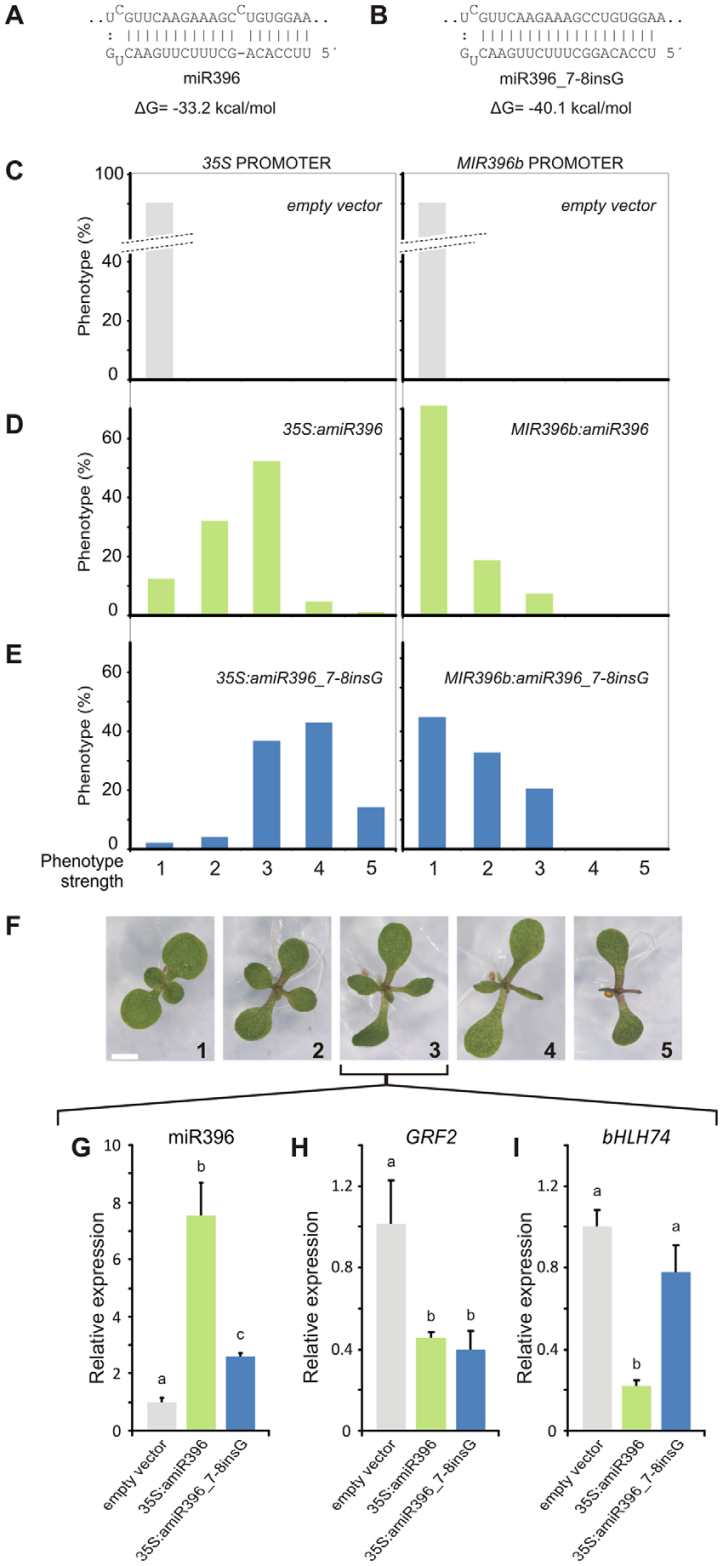
The monocot-version of miR396 is hyperactive towards the *GRFs*. (A,B) Scheme showing the interaction between *Arabidopsis GRF2* and either miR396a (A) or rice miR396e,f (miR396_7-8insG) (B), which is a monocot-specific variant. (C–E) Phenotype frequency in independent transgenic plants (T1) expressing an empty vector (C), *Arabidopsis* miR396a (D), rice miR396e,f (miR396_7-8insG) (E). MiR396a and miR396_7-8insG were expressed from the viral *35S* (left) and *MIR396b* (right) promoters. At least 100 independent transgenic plants were scored for each vector. (F) Phenotypes of transgenic plants (12-day old seedlings) harboring the different vectors. Phenotypes were classified according to the area reduction in leaves #1 and #2. Scale Bar: 2 mm. (G) to (I) miR396, *GRF2* and *bHLH74* levels in control plants (transformed with an empty vector, gray) and transgenic plants overexpressing *Arabidopsis* miR396a (light green) or miR396_7-8insG (blue) displaying an intermediate phenotype. RNA levels were determined by RT-qPCR using pools of 20 independent T1 seedlings. Data shown are mean ± SEM of 3 biological replicates. Bars marked with different letters are significantly different as determined by ANOVA and Duncan's multiple range test (P<0.05).

Recognition of the *GRF* target site by miR396 generates a bulge between positions 7 and 8 of the miRNA (Figure 6A). The insertion of one nucleotide in the monocot-specific version of miR396 eliminates this bulge, strengthening the interaction of the miRNA-target pair by 7 kcal/mol (Figure 6A–6B). Since the contribution of a bulge to the activity of a miRNA has not yet been assayed in plants, we decided to explore it in more detail.

For this purpose, we expressed the two versions of miR396 from the *MIR319a* precursor in *Arabidopsis*, which has already been shown as an efficient driver of artificial miRNA sequences [51]. Ectopic expression of the *Arabidopsis* miR396 mature sequence from the *MIR319a* precursor caused smaller and lanceolated leaves (Figure 6C–6F), in a similar way to the overexpression of the endogenous *MIR396* precursor [21], [22]. Expression of the monocot-version of miR396 (miR396_7-8insG) caused stronger effects on the leaf lamina (Figure 6C–6F). We further expressed both miR396 variants from the endogenous *MIR396b* promoter. While an additional copy of the endogenous miR396 sequence caused a minor impact on leaf development, expression of the monocot miR396 version from the *MIR396b* promoter affected leaf development in more than 50% of the independent transgenic plants (Figure 6C–6F). These results show that the miR396 monocot variant is more active *in vivo*.

We then set up an assay to quantify both miR396 variants by RT-qPCR in the same reaction (for details see Figure S4). We focused on transgenic plants with a moderate reduction in leaf lamina (∼60%), which was observed in 52% or 38% of the primary transgenic plants expressing the endogenous or the monocot-specific miR396 sequence from the 35S viral promoter, respectively (Figure 6D–6F). We measured the levels of miR396 in the transgenics over-accumulating the endogenous *Arabidopsis* miRNA and found that it was eight-fold higher compared to wild-type levels (Figure 6G, for a small RNA blot see Figure S5). In contrast, transgenic seedlings with the same phenotype but expressing the monocot-specific miR396 variant displayed only a two-fold increase (Figure 6G).

Analysis of the miR396-regulated gene *GRF2* in seedlings with moderated phenotypes revealed a decrease of its mRNA levels to 40% in both types of transgenic plants (Figure 6H). We also tested the activity of the two miRNA variants on *bHLH74*. While the overexpression of the endogenous miR396 significantly down-regulated *bHLH74* mRNA (approximately 90%), the monocot-specific version had only a minor effect (Figure 6I). Furthermore, *bHLH74* transcript levels were reduced only 25% in plants expressing the highest levels of the monocot-specific version (data not shown). Therefore, this monocot-specific version of miR396 is selectively more efficient towards the *GRFs*. The addition of an extra nucleotide to this variant causes a bulge to be formed on the miRNA side of the *bHLH74*/miR396 pair (see Figure S6). Altogether, these results show that asymmetric bulges located either in the miRNA or in the target dampen the miRNA-guided cleavage reaction.

We also analyzed the activity of the miR396 variant found in *Selaginella*, pine and poplar (Figure 4A) and determined that it caused slightly stronger developmental defects than the wild-type precursor (see Figure S7). A possible explanation to this is that the miR396_7A>G version replaces an interaction between the A-U pair with a stronger G-C pair, causing a concomitant change of more than two kcal/mol in the interaction energy of the miR396/GRF pair (see Figure S7).

### Suboptimal regulation of miR396 targets in *Arabidopsis thaliana*

The previous results show that a single miR396 gradient generates opposing gradients of expression for its targets (Figure 4), and that a perfect match between miR396 and the *GRFs* increase the *in vivo* efficiency of the miRNA (Figure 6). Next, we decided to analyze whether the bulge present in the miR396-*GRF* pair plays a role in patterning the expression of *GRF2* during leaf development in *Arabidopsis*.

**Figure 7:**
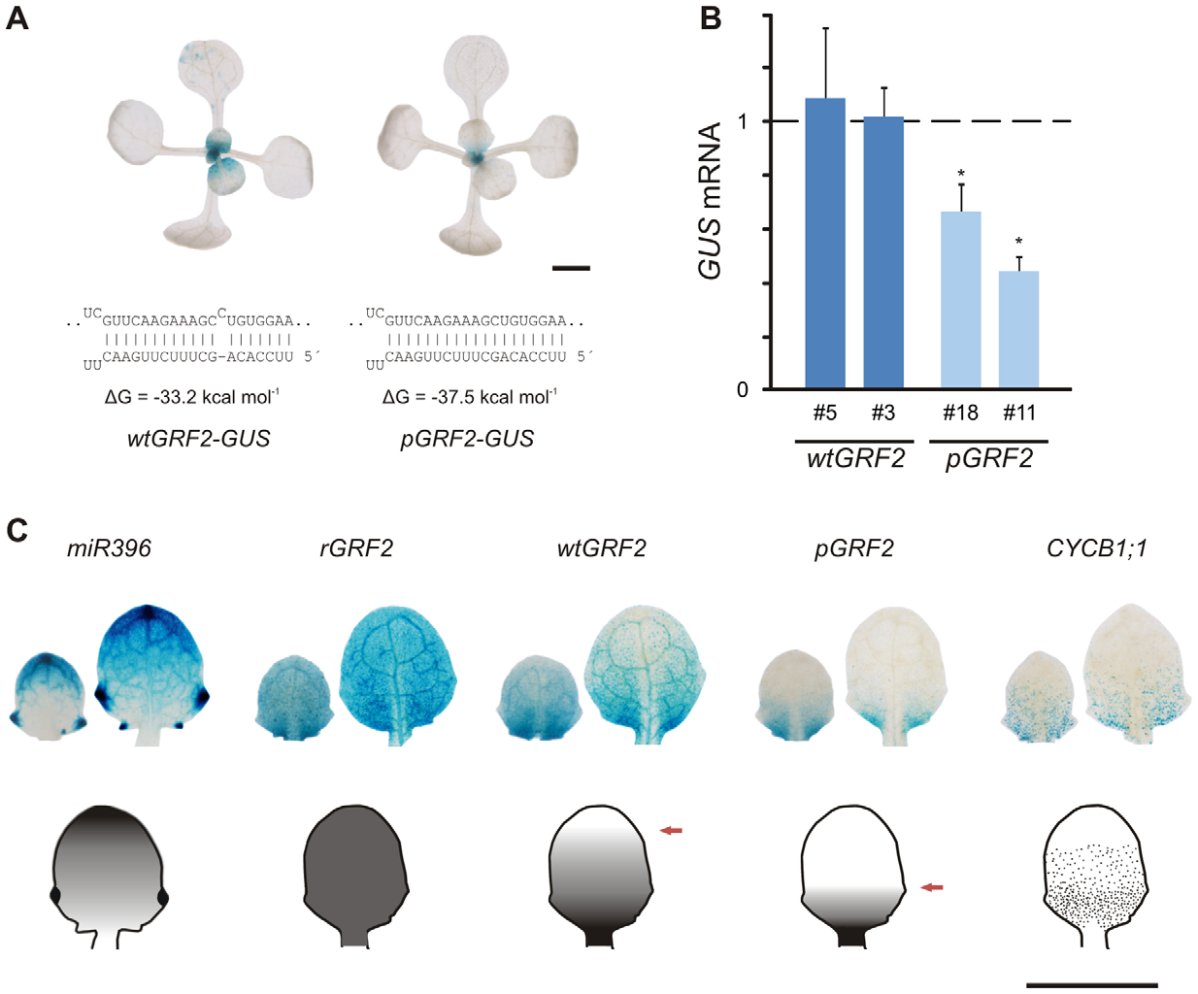
Activity of endogenous miR396 towards different substrates in *Arabidopsis thaliana*. (A) GUS stainings of typical transgenic plants harboring *wtGRF2* and *pGRF2* reporters (15-day old seedlings). Sensors were built by fusing the upstream regulatory regions of *GRF2* and its first 4 exons to *GUS*. The miR396 target site was modified as indicated below the pictures. Interaction energy values for miR396b are indicated below each miRNA-target pair. Scale Bar: 2 mm. (B) Expression levels of *GRF2-GUS* RNA in leaves #1 and #2 (15-day old seedlings) of the different sensors. Two representative lines for each vector out of a total of 20 independent lines were selected. Expression levels were normalized to *wtGRF2-GUS* line #3. Data shown are mean ± SEM of 4 biological replicates. Asterisks indicate significant differences between plants harboring different transgenes, as determined by ANOVA (P<0.05). (C) GUS staining in developing leaves #4 (right) and #5 (left) of transgenic plants harboring miR396, *rGRF2*, *wtGRF2*, *pGRF2* and *CYCLIN B1;1* reporters (14-day old seedlings). Scale Bar: 1 mm.

To test this, we designed another *GRF2-GUS* reporter where the bulge was eliminated from the interaction with the endogenous miR396, thus generating a nearly perfect pairing (*pGRF2-GUS*) (Figure 7A; see Table S6). Whole-mount stainings of *wtGRF2-GUS* revealed its expression in young developing leaves of *Arabidopsis* in 17 out of 20 primary transgenics (Figure 7A). The *pGRF2-GUS* construct had a more limited expression and was restricted towards the proximal part of the leaf. This typical expression pattern was observed in 15 out of 20 transgenic plants for *pGRF2-GUS* (Figure 7A). The remaining reporter lines displayed even weaker levels of GUS staining.

We then measured the *GUS* mRNA in two representative lines for each vector and observed that bulge removal from the *GRF2* reporter caused approximately a two-fold reduction in *GUS* RNA, confirming its higher sensitivity to miR396 (Figure 7B). These results further support our previous findings, *i.e.* the monocot-specific version of miR396, which does not have a bulge in the miRNA-target pair, has a higher activity towards the *GRFs* than the one from *Arabidopsis*.

It has been shown that *GRF2* is expressed in the proximal part of the leaf, which contains proliferating cells [22]. We then compared the expression pattern of several reporters in developing leaves of *Arabidopsis thaliana*. We observed that *MIR396b* has an expression gradient opposite to that of *wtGRF2-GUS* and *pGRF2-GUS* expression (Figure 7C). However, the shape of the latter two gradients is different, being *pGRF2-GUS* tapered off faster than *wtGRF2-GUS*. As expected, *rGRF2-GUS* was expressed throughout the leaf (Figure 7C).

We also stained a *CYCLINB1;1* reporter to identify proliferating cells in similar organs. Comparison of the expression patterns for *GRF2* reporters revealed that only the one harboring the wild-type target sequence was co-expressed with the proliferating cells (Figure 7C). Altogether, these results suggest that particular miRNA-target interactions, which are not a perfect match and therefore likely operate at sub-optimal activity, might have biological implications.

## Discussion

### Acquisition of new targets by ancient miRNAs

Most of the conserved miRNAs regulate transcription factors involved in development and hormone signaling. These target genes generally contain similarly conserved miRNA binding-sites. Studies performed in *Arabidopsis thaliana* and other species have shown that interfering with the regulation of conserved targets by changes in the recognition sites, mutations in the miRNAs [reviewed in [1], [2]] or expressing miRNA-target mimics [12] usually lead to important developmental defects.

Genome-wide analyses of miRNA targets have revealed that ancient miRNAs can regulate targets that are not broadly conserved [36], [37]. However, detection of miRNA-guided cleavage is not necessarily indicative of a relevant biological function. This has already been pointed out for young miRNAs, whose biological functions remain unclear, even though their activities can be detected *in vivo* [9], [11]. The results presented here show that the regulation of *bHLH74* by miR396 has a meaningful impact on its RNA levels, in a similar way to that observed for the widely distributed *GRF* transcription factors. Interestingly, both targets are involved in leaf development. While the *GRFs* control cell number [21], [22], [24], [26], [28], we found that *bHLH74* regulates the vein patterning of the leaf. Furthermore, *GRF2* [22] and *bHLH74* transgenes harboring silent mutations in their miRNA target sites affect leaf development, suggesting that the regulation of both type of targets by miR396 is important for *Arabidopsis* development.

*GRF* regulation by miR396 can be traced back to at least the gymnosperms based on the existence of *GRF* transcription factors with a miRNA target site. In contrast, we could only find *bHLH74* homologues with miR396-binding sites in species within the sister families *Cleomaceae* and *Brassicaceae.* However, it is tempting to speculate that the conservation of a miRNA-target sequence in related species might still be significant and could serve as a tool to identify additional miRNA targets whose regulation might be of biological relevance.

### Subfunctionalization of miRNA family members

Ancient miRNAs are usually found as small gene families, encoding small RNAs of similar or identical sequences. One of the advantages of having families with multiple miRNA members is to provide flexibility in the way miRNAs are themselves regulated [52]. Additionally, differences in the miRNA sequences could cause related miRNAs to regulate different sets of targets. This has been previously shown for the miRNAs miR319 and miR159, which are similar in sequence but still regulate different genes [16], [17], [18], [19]. While miR319 can guide *TCP* and *MYB* genes to cleavage, specific base differences prevent miR159 activity on the *TCPs* [17].

The results reported here suggest that similar sequences could have also acquired specialized functions to regulate the same set of targets with different efficiency. The conserved interaction between miR396 and the *GRFs* has a bulge at position 7–8 of the target site. Removal of this bulge by either the addition of one base to the miRNA, as seen in the monocot-specific miRNA variant, or by the removal of a base from the *GRF* target site, results in a higher miRNA activity. Interestingly, the addition of a base in the *Arabidopsis* miR396 selectively improves its efficiency towards the *GRFs* at the expense of losing activity towards *bHLH74*, suggesting that bulges in miRNA/target pairs could be used for differential target regulation in miRNA networks.

Our systematic analysis of evolutionary conserved plant miRNAs has shown that additional variations exist in other miRNA sequences, including changes in the 5′ sensitive region. It might be interesting to further explore the occurrence in nature of additional examples of miRNA specialization, and whether they cause changes in target specificity and/or efficiency.

It has been proposed that *Arabidopsis* miR396 contributes to the fine-tuning of *GRF* expression [22]. Here, we have further shown that only a *GRF2* reporter under suboptimal regulation by the endogenous miR396 can overlap the proliferative region of a developing leaf in *Arabidopsis thaliana*. It is plausible, however, that a gross down-regulation of the *GRFs* under specific conditions or in specific cells, such as the one caused by the monocot miR396 variant, could also be advantageous.

### Multiple target regulation by miR396 in *Arabidopsis thaliana*

MiR396 is expressed at low levels in the meristem and leaf primordia, and then it steadily accumulates as leaves develop [22]. When considering a single developing organ, miR396 accumulates in the more mature and distal part, with a miRNA gradient proceeding towards the base of the organ.

Analysis of *bHLH74* and *GRF2* expression patterns revealed that they both shared a temporal component, which is imposed by the accumulation of miR396 during leaf development. Both genes are expressed in young organs as a consequence of miR396 activity. Still, *bHLH74* and *GRF2* reporters do not have identical expression patterns, as the *bHLH* is more restricted to the vasculature while the *GRF* is more widely distributed throughout the leaf mesophyll cells. The exact tissue of expression might be governed by *cis* regulatory sequences in the promoters of the different targets. Therefore, several layers of regulation can ultimately generate unique and coordinated expression patterns on target genes belonging to the same miRNA regulatory network.

Another potential level of complexity in the regulation of different targets by miR396 might arise from the miRNA expression gradient in a developing leaf, which extends from the distal part of the organ towards its base. In principle, a single miRNA gradient can generate different expression gradients of its targets, depending at least partially on the exact nature of the miRNA-target pair.

## Materials and Methods

### Plant material and leaf analysis

*Arabidopsis* ecotype Col-0 was used for all experiments, with the exception of the *GRF2-GUS* and *bHLH74-GUS* reporters, which were analyzed in *rdr6* background. Plants were grown in long (16 h light/8 h dark) or short photoperiods (8 h light/16 h dark) at 23°C.

To analyze the vein pattern, leaves were fixed with FAA and cleared with a chloral hydrate solution. Pictures were then taken under dark field illumination in a dissecting microscope. The number of branching points (NBP) [43] was measured per leaf half in Figure 2D, and in the whole leaf in Figure 3D.

### Transgenes

See Table S6 for a list of binary plasmids used in this study. The miRNA target motif in *GRF2* and *bHLH74* was altered introducing synonymous mutations in a cloned wild-type genomic fragment using the QuikChange Site Directed Mutagenesis Kit (Stratagene). Artificial miRNAs [51] were generated by PCR, and *MIM396* was generated by gene synthesis (Mr. Gene GmbH).

### Expression analysis

RNA was extracted using TRIzol reagent (Invitrogen) and 1,0 µg of total RNA was treated with RQ1 RNase-free Dnase (Promega). Next, first-strand cDNA synthesis was carried out using SuperScriptTM III Reverse Transcriptase (Invitrogen) with the appropriate primers. PCR reactions were performed in a Mastercycler ep realplex thermal cycler (Eppendorf) using SYBRGreen I (Roche) to monitor dsDNA synthesis. MiR396 and miR396_7-8insG levels were concurrently determined in each sample by stem-loop RT-qPCR [53]. A scheme of the strategy used for the simultaneous quantification of miR396 and miR396_7-8insG is provided in Figure S4. Relative transcript level was determined for each sample, normalized using *PROTEIN PHOSPHATASE 2A* (AT1G13320) cDNA levels [54]. MiR396 levels were also estimated by small RNA blots as described previously [22]. Primer sequences are given in Table S7.

To visualize reporter activity, transgenic plants were subjected to GUS staining, as described previously [55]. RNA adaptor ligation, reverse transcription and 5′RACE were performed according to the procedure described previously in order to determine RNA degradation products [56].

### Sequence analysis

Small RNA sequences obtained from miRBase (17.0) [4] were used for analyses of miRNA sequence variations in conserved miRNA families in angiosperms [9]. A consensus sequence was identified for each family and deviations from the consensus at each position were quantified. The number of variations was normalized to the total number of miRNA family members, so that each family contributed equally.

### Accession numbers

A list of relevant AGI locus identifiers is provided in Table S7.

## Supporting Information

Figure S1 Sequence alignment of *bHLH74* homologs from several species. Alignment of partial coding sequences for *bHLH74*. A red box highlights the miR396 target site and a grey box depicts part of the coding sequence of the bHLH domain. Conserved positions across all species are indicated by asterisks. See Table S4 for accession numbers of sequences used.
(TIF)

Figure S2 Effects of high expression levels of *bHLH74* on *Arabidopsis thaliana* development. (A) Schematic representation of the *35S:bHLH74* and *35S:rbHLH74* constructs. (B) Phenotypes observed in 12-day old T1 seedlings overexpressing *bHLH74* or *rbHLH74*. Phenotypes were classified according to their strength (numbers 1 to 4). Arrowheads indicate the elongated cotyledons observed only in *35S:rbHLH74* seedlings. Scale Bar: 2 mm. (C) Phenotype frequencies, according to panel (B), observed in at least 100 independent T1 plants expressing each vector.
(TIF)

Figure S3 Variations in the mature sequence of conserved miRNA families. (A) and (B) Variations in the mature sequence of miRNAs conserved in angiosperms (20 families). Bars represent the nucleotide changes in miRNA obtained for each family from position 1 to 21. Variation for each family was normalized to the number of members so that each family contributes equally. MiRNAs miR159 and miR319 were considered as a single family. (A) Variations in *Arabidopsis thaliana* (88 miRNAs). (B) Plant mature sequences (miRBase 16.0) belonging to 42 species. A black bar in position 8 (highlighted with an asterisk) represents the contribution of the miR396 variants.
(TIF)

Figure S4 Description of the method used to quantify miR396 variants. The retrotranscription reaction was performed using a stem-loop oligo that matches the three miR396 variants. For the qPCR, an equimolar mix of primers matching the miR396 variants was used. PCR efficiencies were checked to be equivalent for the different miRNAs.
(TIF)

Figure S5 Small RNA blot of miR396. Small RNA blot showing miR396 levels in control plants (transformed with an empty vector) and transgenic plants expressing *Arabidopsis* miR396b or miR396_7-8insG displaying an intermediate phenotype (see Figure 6F). A locked nucleic acid (LNA) probe against miR396b was used.
(TIF)

Figure S6 Interaction of the *bHLH74* target site with (A) *Arabidopsis* miR396a and (B) the monocot-specific variant (miR396_7-8insG).
(TIF)

Figure S7 Overexpression of miR396_7A>G in *Arabidopsis thaliana*. (A) Scheme showing the secondary structure of the miRNA-miRNA* region in miR396 precursors from *Arabidopsis thaliana*, *Selaginella moellendorffii* and *Picea glauca*. miR396 sequence is indicated in red. Note that a G-A change in position 7 of the mature miRNA sequence (indicated in light gray) does not alter the secondary structure of the precursors. (B) and (C) Diagram showing the interaction between *Arabidopsis GRF2* and miR396b (B) or the variant found in pine and poplar (C). (D) and (E) Phenotypes of independent transgenic seedlings overexpressing miR396b (D) or the miR396b_7A>G (E) variants. Phenotypes were classified as wild type, medium and strong which correspond to the first, third and fifth picture from the left in Figure 6F. At least 100 independent plants were scored for each vector.
(TIF)

Table S1 Predicted targets of miR396 in *Arabidopsis thaliana*.
(DOC)

Table S2 Predicted targets of miR396 in poplar.
(DOC)

Table S3 Predicted targets of miR396 in rice.
(DOC)

Table S4 Sequences used to analyze the conservation of the miR396 target site.
(DOC)

Table S5 Expression of different miR396 variants in publicly available small RNA sequencing libraries.
(DOC)

Table S6 Binary plasmids prepared for this study.
(DOC)

Table S7 Relevant locus identifiers, mutant alleles and RT-qPCR primers.
(DOC)
