# Supplementary figures and images for: Functional Specialization of the Plant miR396 Regulatory Network through Distinct MicroRNA–Target Interactions
Source: PLoS Genet. 2012 Jan 5;8(1):e1002419. doi: 10.1371/journal.pgen.1002419 (PMC3252272; doi:10.1371/journal.pgen.1002419)

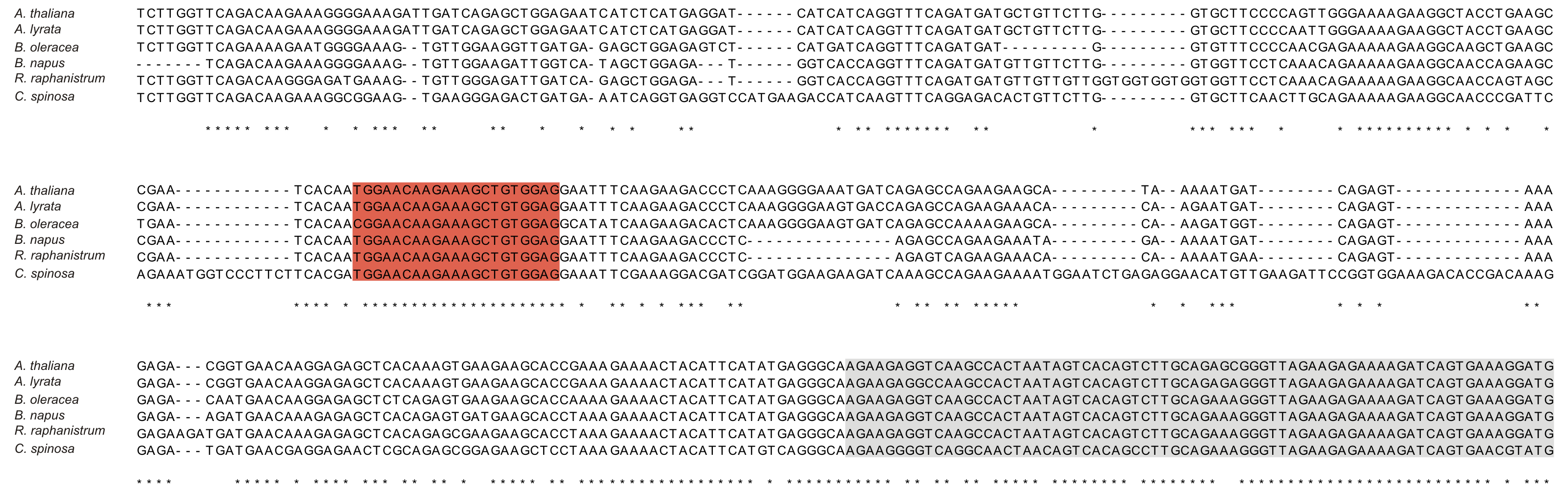

Supplement: Figure S1 — Sequence alignment of bHLH74 homologs from several species. Alignment of partial coding sequences for bHLH74. A red box highlights the miR396 target site and a grey box depicts part of the coding sequence of the bHLH domain. Conserved positions across all species are indicated by asterisks. See Table S4 for accession numbers of sequences used. (TIF) [file pgen.1002419.s001.tif]

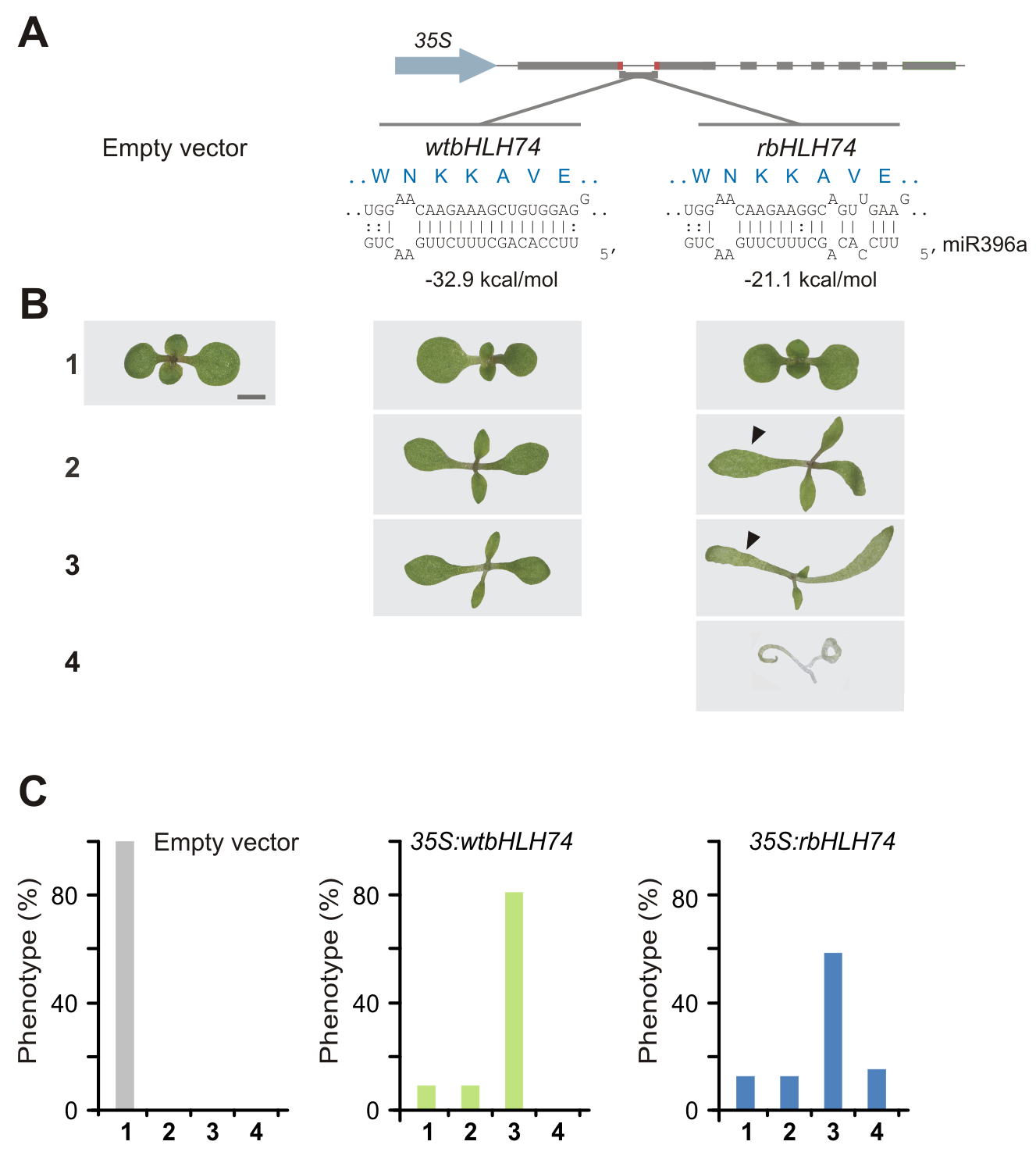

Supplement: Figure S2 — Effects of high expression levels of bHLH74 on Arabidopsis thaliana development. (A) Schematic representation of the 35S:bHLH74 and 35S:rbHLH74 constructs. (B) Phenotypes observed in 12-day old T1 seedlings overexpressing bHLH74 or rbHLH74. Phenotypes were classified according to their strength (numbers 1 to 4). Arrowheads indicate the elongated cotyledons observed only in 35S:rbHLH74 seedlings. Scale Bar: 2 mm. (C) Phenotype frequencies, according to panel (B), observed in at least 100 independent T1 plants expressing each vector. (TIF) [file pgen.1002419.s002.tif]

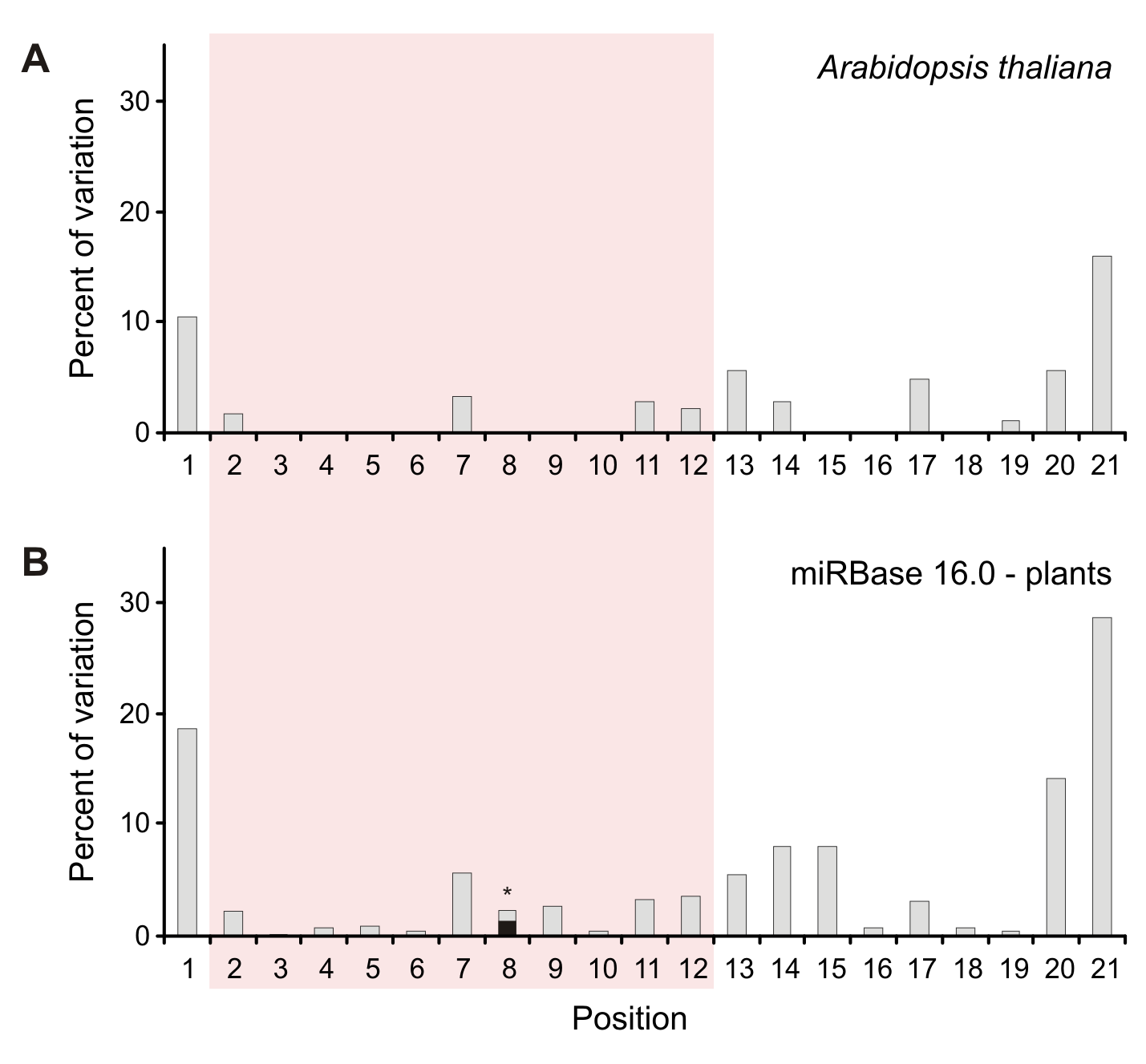

Supplement: Figure S3 — Variations in the mature sequence of conserved miRNA families. (A) and (B) Variations in the mature sequence of miRNAs conserved in angiosperms (20 families). Bars represent the nucleotide changes in miRNA obtained for each family from position 1 to 21. Variation for each family was normalized to the number of members so that each family contributes equally. MiRNAs miR159 and miR319 were considered as a single family. (A) Variations in Arabidopsis thaliana (88 miRNAs). (B) Plant mature sequences (miRBase 16.0) belonging to 42 species. A black bar in position 8 (highlighted with an asterisk) represents the contribution of the miR396 variants. (TIF) [file pgen.1002419.s003.tif]

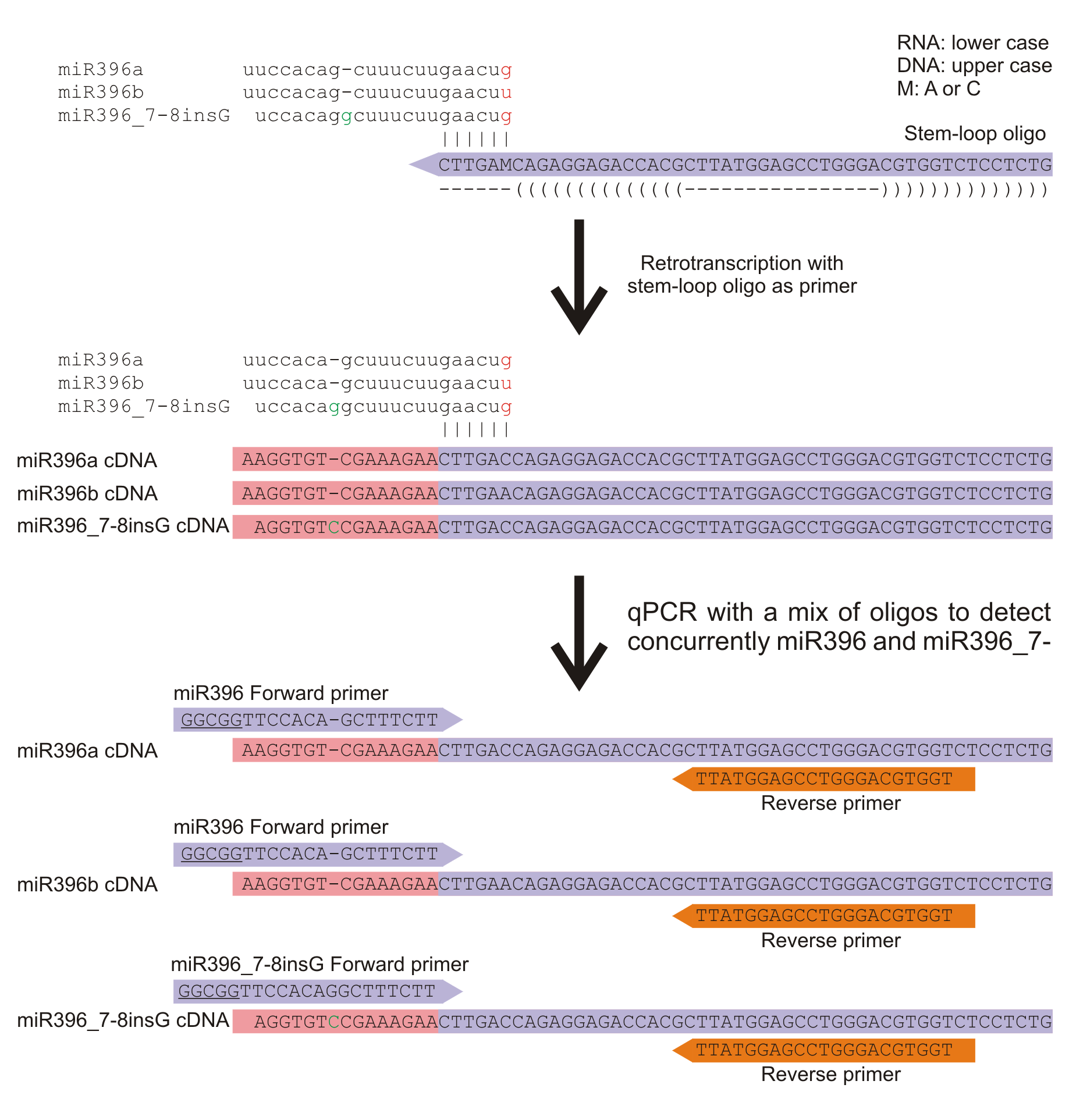

Supplement: Figure S4 — Description of the method used to quantify miR396 variants. The retrotranscription reaction was performed using a stem-loop oligo that matches the three miR396 variants. For the qPCR, an equimolar mix of primers matching the miR396 variants was used. PCR efficiencies were checked to be equivalent for the different miRNAs. (TIF) [file pgen.1002419.s004.tif]

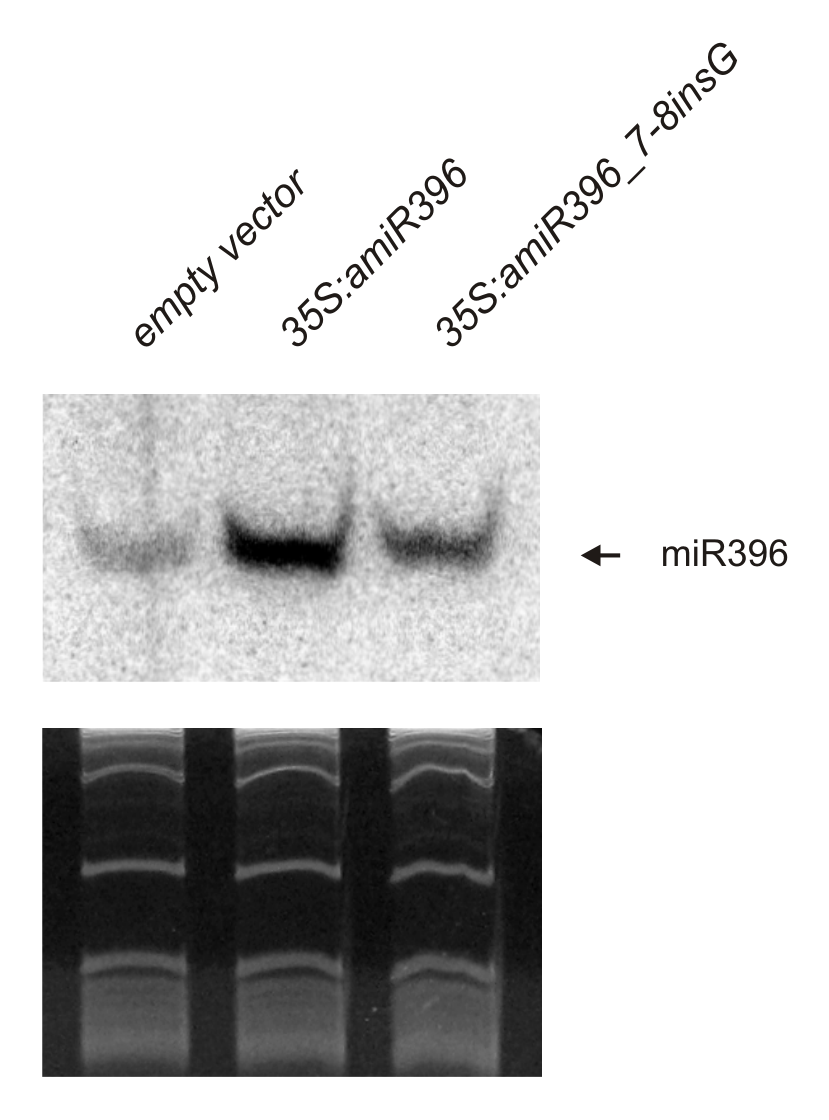

Supplement: Figure S5 — Small RNA blot of miR396. Small RNA blot showing miR396 levels in control plants (transformed with an empty vector) and transgenic plants expressing Arabidopsis miR396b or miR396_7-8insG displaying an intermediate phenotype (see Figure 6F). A locked nucleic acid (LNA) probe against miR396b was used. (TIF) [file pgen.1002419.s005.tif]

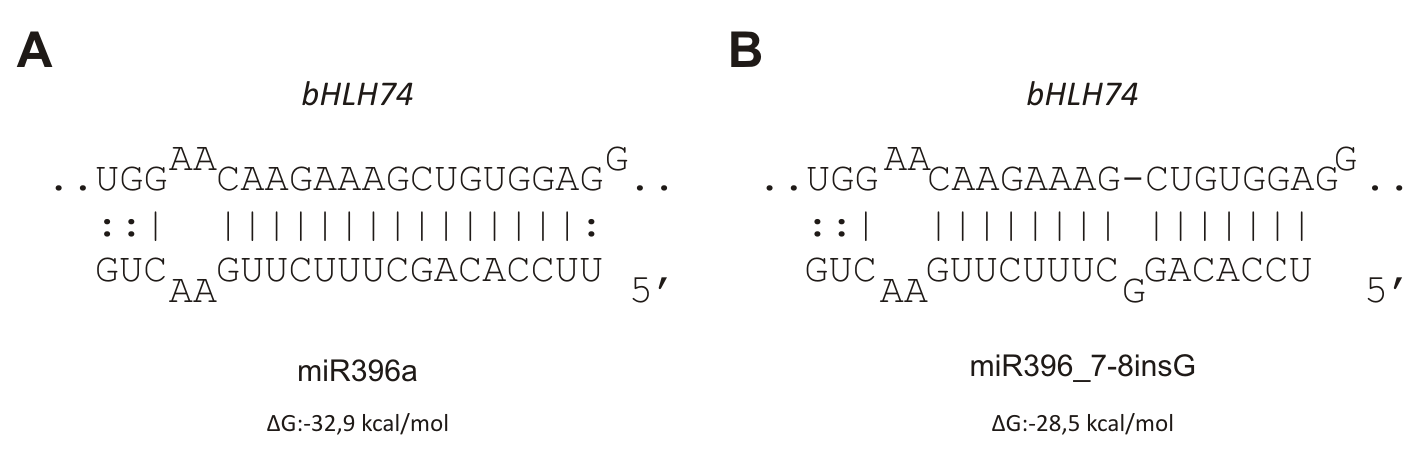

Supplement: Figure S6 — Interaction of the bHLH74 target site with (A) Arabidopsis miR396a and (B) the monocot-specific variant (miR396_7-8insG). (TIF) [file pgen.1002419.s006.tif]

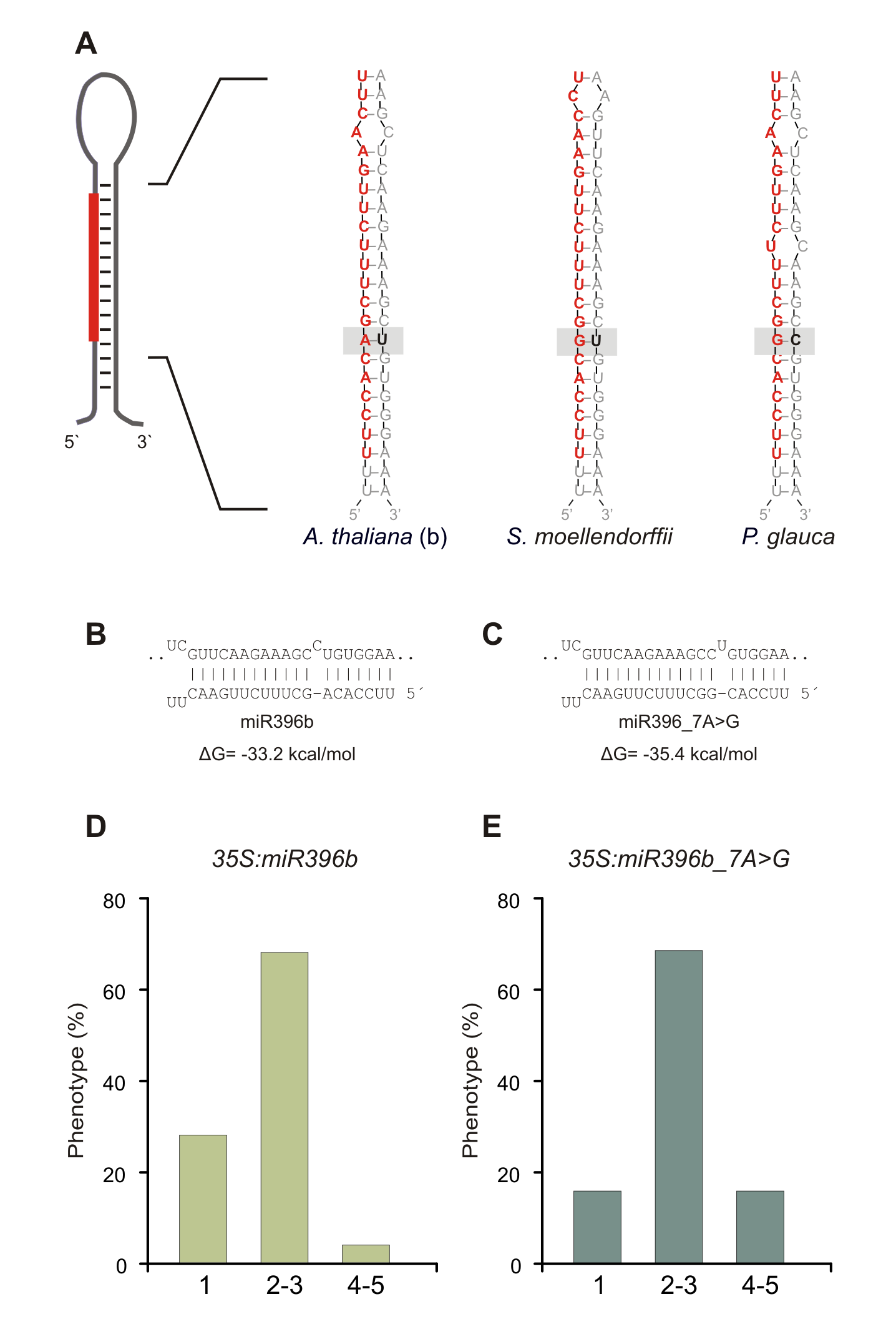

Supplement: Figure S7 — Overexpression of miR396_7A>G in Arabidopsis thaliana. (A) Scheme showing the secondary structure of the miRNA-miRNA* region in miR396 precursors from Arabidopsis thaliana, Selaginella moellendorffii and Picea glauca. miR396 sequence is indicated in red. Note that a G-A change in position 7 of the mature miRNA sequence (indicated in light gray) does not alter the secondary structure of the precursors. (B) and (C) Diagram showing the interaction between Arabidopsis GRF2 and miR396b (B) or the variant found in pine and poplar (C). (D) and (E) Phenotypes of independent transgenic seedlings overexpressing miR396b (D) or the miR396b_7A>G (E) variants. Phenotypes were classified as wild type, medium and strong which correspond to the first, third and fifth picture from the left in Figure 6F. At least 100 independent plants were scored for each vector. (TIF) [file pgen.1002419.s007.tif]
