# Supplementary material for: Functional Specialization of the Plant miR396 Regulatory Network through Distinct MicroRNA–Target Interactions
Source: PLoS Genet. 2012 Jan 5;8(1):e1002419. doi: 10.1371/journal.pgen.1002419 (PMC3252272; doi:10.1371/journal.pgen.1002419)
Supplement: Table S1 — Predicted targets of miR396 in Arabidopsis thaliana. (DOC) [file pgen.1002419.s008.doc]

**Table S1. Predicted targets of miR396 in *Arabidopsis thaliana***.

|  |  |  |  | Relative expression level | | |
| --- | --- | --- | --- | --- | --- | --- |
| Predicted target |  | Description | ∆G | *dcl1* | *hyl1* | *se* |
| AT2G22840 |  | AtGRF1 (GROWTH-REGULATING FACTOR 1) | -35.78kcal/mol | 1.9 | 1.5 | 2.7 |
| AT2G45480 |  | AtGRF9 (GROWTH-REGULATING FACTOR 9) | -35.78kcal/mol | nd | nd | nd |
| AT2G36400 |  | AtGRF3 (GROWTH-REGULATING FACTOR 3) | -35.78kcal/mol | 1.3 | 1.3 | 2.1 |
| AT3G52910 |  | AtGRF4 (GROWTH-REGULATING FACTOR 4) | -35.78kcal/mol | nd | nd | nd |
| AT4G24150 |  | AtGRF8 (GROWTH-REGULATING FACTOR 8) | -35.78kcal/mol | 1.1 | 1.3 | 8.6 |
| AT4G37740 |  | AtGRF2 (GROWTH-REGULATING FACTOR 2) | -35.78kcal/mol | 1.8 | 1.3 | 1.8 |
| AT5G53660 |  | AtGRF7 (GROWTH-REGULATING FACTOR 7) | -35.78kcal/mol | 1.0 | 0.8 | 1.5 |
| AT1G80260 |  | EMB1427 (EMBRYO DEFECTIVE 1427); tubulin binding | -35.55kcal/mol | nd | nd | nd |
| AT1G20570 |  | tubulin family protein | -35.55kcal/mol | 1.1 | 1.1 | 0.8 |
| AT1G10120 |  | DNA binding / transcription factor | -33.21kcal/mol | 1.3 | 1.8 | 1.5 |
| AT5G04810 |  | pentatricopeptide (PPR) repeat-containing protein | -31.47kcal/mol | 0.9 | 0.9 | 1.0 |
| AT1G60140 |  | ATTPS10 (Arabidopsis thaliana trehalose phosphatase/synthase 10); transferase, transferring glycosyl groups / trehalose-phosphatase | -31.25kcal/mol | 0.8 | 0.9 | 1.1 |
| AT2G38823 |  | unknown protein | -31.12kcal/mol | nd | nd | nd |
| AT2G39360 |  | protein kinase family protein | -31.09kcal/mol | 1.3 | 1.2 | 0.7 |
| AT3G12230 |  | SCPL14 (serine carboxypeptidase-like 14); serine carboxypeptidase | -31.07kcal/mol | 1.1 | 1.0 | 2.2 |
| AT2G35040 |  | AICARFT/IMPCHase bienzyme family protein | -30.91kcal/mol | 1.2 | 1.0 | 0.9 |
| AT5G24660 |  | unknown protein | -30.10kcal/mol | 3.8 | 1.9 | 0.9 |
| AT1G23580 |  | unknown protein | -29.93kcal/mol | 0.4 | 0.6 | 3.0 |
| AT1G23600 |  | unknown protein | -29.93kcal/mol | 0.3 | 0.7 | 0.9 |
| AT1G48380 |  | RHL1 (ROOT HAIRLESS 1),unknown protein | -29.85kcal/mol | 1.3 | 1.0 | 1.1 |
| AT1G01520 |  | myb family transcription factor | -29.84kcal/mol | 1.2 | 0.2 | 1.6 |
| AT1G52620 |  | pentatricopeptide (PPR) repeat-containing protein | -28.98kcal/mol | 1.1 | 1.2 | 1.1 |
| AT5G04400 |  | ANAC077 (Arabidopsis NAC domain containing protein 77); DNA binding | -28.97kcal/mol | 1.7 | 1.1 | 1.1 |
| AT1G13140 |  | CYP86C3 (cytochrome P450, family 86, subfamily C, polypeptide 3); oxygen binding | -28.86kcal/mol | 0.4 | 0.8 | 0.9 |

Putative miR396 targets were predicted by WMD3 target search tool ([http://wmd3.weigelworld.org/](http://wmd3.weigelworld.org/cgi-in/webapp.cgi?page=TargetSearch;project=stdwmd); WMD3 TAIR9_cdna_20090619). Genes were sorted according to the ∆G value. The relative expression levels in miRNA mutants were obtained from genevestigator ([www.genevestigator.com](http://www.genevestigator.com/)). *dcl1* and *hyl1* data, [Allen EM](http://www.ncbi.nlm.nih.gov/sites/entrez?db=PubMed&term=Allen EM%5BAuthor%5D), et al., Cell 2005; *se* data, Lobbes D. et al., EMBO 2006. *GRFs* are indicated in green. Putative miR396 targets with an increase in their expression levels of at least 30% in two mutants are highlighted in light blue.
