# Supplementary material for: Functional Specialization of the Plant miR396 Regulatory Network through Distinct MicroRNA–Target Interactions
Source: PLoS Genet. 2012 Jan 5;8(1):e1002419. doi: 10.1371/journal.pgen.1002419 (PMC3252272; doi:10.1371/journal.pgen.1002419)
Supplement: Table S2 — Predicted targets of miR396 in poplar. (DOC) [file pgen.1002419.s009.doc]

**Table S2. Predicted targets of miR396 in poplar.**

|  |  |  | |  | | Variants | | |
| --- | --- | --- | --- | --- | --- | --- | --- | --- |
| Predicted target | Best hit in *Arabidopsis* | Description of the *Arabidopsis* hit |  | | a,b,c,d,e | | f,g |  |
| POPTR_0007s14720 | AT4G37740 | AtGRF2 (GROWTH-REGULATING FACTOR 2) |  | |  | |  |  |
| POPTR_0014s01320 | AT4G37740 | AtGRF2 (GROWTH-REGULATING FACTOR 2) |  | |  | |  |  |
| POPTR_0001s16920 | AT3G13960 | AtGRF5 (GROWTH-REGULATING FACTOR 5) |  | |  | |  |  |
| POPTR_0014s00780 | AT4G37740 | AtGRF2 (GROWTH-REGULATING FACTOR 2) |  | |  | |  |  |
| POPTR_0001s11480 | AT4G24150 | AtGRF8 (GROWTH-REGULATING FACTOR 8) |  | |  | |  |  |
| POPTR_0013s07500 | AT2G36400 | AtGRF3 (GROWTH-REGULATING FACTOR 3) |  | |  | |  |  |
| POPTR_0003s10000 | AT2G22840 | AtGRF1 (GROWTH-REGULATING FACTOR 1) |  | |  | |  |  |
| POPTR_0006s14560 | AT2G36400 | AtGRF3 (GROWTH-REGULATING FACTOR 3) |  | |  | |  |  |
| POPTR_0001s01480 | AT4G37740 | AtGRF2 (GROWTH-REGULATING FACTOR 2) |  | |  | |  |  |
| POPTR_0003s06360 | AT3G13960 | AtGRF5 (GROWTH-REGULATING FACTOR 5) |  | |  | |  |  |
| POPTR_0018s07140 | AT3G13960 | AtGRF5 (GROWTH-REGULATING FACTOR 5) |  | |  | |  |  |
| POPTR_0002s11590 | AT4G37740 | AtGRF2 (GROWTH-REGULATING FACTOR 2) |  | |  | |  |  |
| POPTR_0006s11630 | AT3G52910 | AtGRF4 (GROWTH-REGULATING FACTOR 4) |  | |  | |  |  |
| POPTR_0001s08310 | AT5G53660 | AtGRF7 (GROWTH-REGULATING FACTOR 7) |  | |  | |  |  |
| POPTR_0014s06750 | AT2G45480 | AtGRF9 (GROWTH-REGULATING FACTOR 9) |  | |  | |  |  |
| POPTR_0015s00860 | AT5G53660 | AtGRF7 (GROWTH-REGULATING FACTOR 7) |  | |  | |  |  |
| POPTR_0012s02950 | AT5G53660 | AtGRF7 (GROWTH-REGULATING FACTOR 7) |  | |  | |  |  |
| POPTR_0019s05910 | AT2G36400 | AtGRF3 (GROWTH-REGULATING FACTOR 3) |  | |  | |  |  |
| POPTR_0008s19510 | AT1G59640 | ZCW32 (BIGPETAL, BIGPETALUB); DNA binding / transcription factor |  | |  | |  |  |
| POPTR_0010s04920 | AT1G59640 | ZCW32 (BIGPETAL, BIGPETALUB); DNA binding / transcription factor |  | |  | |  |  |
| POPTR_0014s11190 | AT4G32450 | pentatricopeptide (PPR) repeat-containing protein |  | |  | |  |  |
| POPTR_0019s08970 | AT5G05180 | unknown protein |  | |  | |  |  |
| POPTR_0003s11580 | AT4G22760 | pentatricopeptide (PPR) repeat-containing protein |  | |  | |  |  |
| POPTR_0001s18810 | AT1G34050 | ankyrin repeat family protein |  | |  | |  |  |
| POPTR_0014s07310 | AT1G01860 | PFC1 (PALEFACE 1) |  | |  | |  |  |
| POPTR_0016s11940 | AT3G54140 | proton-dependent oligopeptide transport (POT) family protein |  | |  | |  |  |
| POPTR_0007s14300 | AT4G24540 | AGL24 (AGAMOUS-LIKE 24); transcription factor |  | |  | |  |  |
| POPTR_0007s01770 | AT3G59530 | strictosidine synthase family protein |  | |  | |  |  |
| POPTR_0017s05630 | AT3G59530 | strictosidine synthase family protein |  | |  | |  |  |
| POPTR_0010s02680 | AT3G06320 | ribosomal protein L33 family protein |  | |  | |  |  |
| POPTR_0005s14780 | AT5G37970 | S-adenosyl-L-methionine:carboxyl methyltransferase family protein |  | |  | |  |  |
| POPTR_0005s14810 | AT5G37970 | S-adenosyl-L-methionine:carboxyl methyltransferase family protein |  | |  | |  |  |
| POPTR_1263s00200 | AT5G37970 | S-adenosyl-L-methionine:carboxyl methyltransferase family protein |  | |  | |  |  |
| POPTR_0006s20610 | AT3G57990 | unknown protein |  | |  | |  |  |
| POPTR_0006s26980 | AT3G49500 | RDR6 (RNA-DEPENDENT RNA POLYMERASE 6); nucleic acid binding |  | |  | |  |  |
| POPTR_0008s04690 | AT3G55060 | unknown protein |  | |  | |  |  |
| POPTR_0006s10470 | AT5G17840 | chaperone protein dnaJ-related |  | |  | |  |  |
| POPTR_0030s00470 | AT3G04920 | 40S ribosomal protein S24 (RPS24A) |  | |  | |  |  |
| POPTR_0018s11750 | AT5G58000 | CPL4 (C-TERMINAL DOMAIN PHOSPHATASE-LIKE 4) |  | |  | |  |  |
| POPTR_0010s16350 | AT4G15215 | ATPDR13/PDR13 (PLEIOTROPIC DRUG RESISTANCE 13); ATP binding / ATPase/ nucleoside-triphosphatase/ nucleotide binding |  | |  | |  |  |
| POPTR_0018s07330 | AT1G53920 | GLIP5 (GDSL-motif lipase 5); carboxylic ester hydrolase |  | |  | |  |  |
| POPTR_0011s12250 | AT3G15430 | regulator of chromosome condensation (RCC1) family protein |  | |  | |  |  |
| POPTR_0015s07150 | AT2G36640 | ATECP63 (EMBRYONIC CELL PROTEIN 63) |  | |  | |  |  |
| POPTR_0001s00600 | AT4G27500 | PPI1 (PROTON PUMP INTERACTOR 1) |  | |  | |  |  |

Putative miR396 targets were predicted by the WMD3 target search tool (<http://wmd3.weigelworld.org/>; WMD3 Populus trichocarpa v2.0). For each candidate gene, the best blast hit in *Arabidopsis* is shown. *GRFs* are indicated in green. Color bars (red, orange and yellow) indicate the members of the miR396 family that are predicted to target each gene.
