# Supplementary material for: Functional Specialization of the Plant miR396 Regulatory Network through Distinct MicroRNA–Target Interactions
Source: PLoS Genet. 2012 Jan 5;8(1):e1002419. doi: 10.1371/journal.pgen.1002419 (PMC3252272; doi:10.1371/journal.pgen.1002419)
Supplement: Table S3 — Predicted targets of miR396 in rice. (DOC) [file pgen.1002419.s010.doc]

**Table S3. Predicted targets of miR396 in rice.**

|  | |  | |  | |  | | Variants | | |
| --- | --- | --- | --- | --- | --- | --- | --- | --- | --- | --- |
| Predicted target | Best hit in *Arabidopsis* | | Description of the *Arabidopsis* hit | |  | | a,b,c | | e,f |  |
| Os11g35030 (*OsGRF8 ^a^*) | AT2G22840 | | AtGRF1 (GROWTH-REGULATING FACTOR 1) | |  | |  | |  |  |
| Os06g10310 (*OsGRF2 ^a^*) | AT2G36400 | | AtGRF3 (GROWTH-REGULATING FACTOR 3) | |  | |  | |  |  |
| Os06g02560 (*OsGRF6 ^a^*) | AT2G36400 | | AtGRF3 (GROWTH-REGULATING FACTOR 3) | |  | |  | |  |  |
| Os04g51190 (*OsGRF3 ^a^*) | AT2G22840 | | AtGRF1 (GROWTH-REGULATING FACTOR 1) | |  | |  | |  |  |
| Os03g51970 (*OsGRF5 ^a^*) | AT4G37740 | | AtGRF2 (GROWTH-REGULATING FACTOR 2) | |  | |  | |  |  |
| Os03g47140 (*OsGRF9 ^a^*) | AT2G22840 | | AtGRF1 (GROWTH-REGULATING FACTOR 1) | |  | |  | |  |  |
| Os02g53690 (*OsGRF1 ^a^)* | AT2G06200 | | AtGRF6 (GROWTH-REGULATING FACTOR 6) | |  | |  | |  |  |
| Os02g47280 (*OsGRF4 ^a^*) | AT2G22840 | | AtGRF1 (GROWTH-REGULATING FACTOR 1) | |  | |  | |  |  |
| Os02g45570 (*OsGRF10 ^a^*) | AT2G36400 | | AtGRF3 (GROWTH-REGULATING FACTOR 3) | |  | |  | |  |  |
| Os12g29980 (*OsGRF7 ^a^*) | AT2G22840 | | AtGRF1 (GROWTH-REGULATING FACTOR 1) | |  | |  | |  |  |
| Os04g24190 | AT5G35660 | | pseudogene | |  | |  | |  |  |
| Os06g28060 | AT1G19100 | | ATP-binding region, ATPase-like domain-containing protein-related | |  | |  | |  |  |
| Os12g05860 | AT5G38910 | | germin-like protein, putative | |  | |  | |  |  |
| Os04g30450 | AT4G13800 | | permease-related | |  | |  | |  |  |
| Os04g49430 | AT1G65920 | | regulator of chromosome condensation (RCC1) family protein / zinc finger protein-related | |  | |  | |  |  |
| Os07g05470 | AT2G34680 | | AIR9 (Auxin-Induced in Root cultures 9); protein binding | |  | |  | |  |  |
| Os04g38810 | AT3G05470 | | formin homology 2 domain-containing protein / FH2 domain-containing protein | |  | |  | |  |  |
| Os01g16530 | AT3G05470 | | formin homology 2 domain-containing protein / FH2 domain-containing protein | |  | |  | |  |  |
| Os04g47040 | AT5G41315 | | GL3 (GLABRA 3); transcription factor | |  | |  | |  |  |
| Os04g48510 (*OsGRF12 ^a^*) | AT2G36400 | | AtGRF3 (GROWTH-REGULATING FACTOR 3) | |  | |  | |  |  |
| Os06g27800 | AT5G66420 | | unknown protein | |  | |  | |  |  |
| Os02g43130 | AT2G21300 | | kinesin motor family protein,microtubule motor | |  | |  | |  |  |
| Os02g43050 | AT2G21300 | | kinesin motor family protein,microtubule motor | |  | |  | |  |  |

Putative miR396 targets predicted with the WMD3 target search tool (<http://wmd3.weigelworld.org/>; WMD3 Rice_TIGR5.fa) in rice. For each candidate gene, the best blast hit in *Arabidopsis* is shown. *GRFs* are indicated in green. Color bars (red, orange and yellow) indicate the members of the miR396 family that are predicted to target each gene.

^a^ Classification according to: Choi D, Kim JH and Kende H (2004). Whole Genome Analysis of the OsGRF Gene Family Encoding Plant-Specific Putative Transcription Activators in Rice (Oryza sativa L.). Plant Cell Physiol. 45(7): 897–904.
