# Supplementary material for: Functional Specialization of the Plant miR396 Regulatory Network through Distinct MicroRNA–Target Interactions
Source: PLoS Genet. 2012 Jan 5;8(1):e1002419. doi: 10.1371/journal.pgen.1002419 (PMC3252272; doi:10.1371/journal.pgen.1002419)
Supplement: Table S4 — Sequences used to analyze the conservation of the miR396 target site. (DOC) [file pgen.1002419.s011.doc]

**Table S4. Sequences used to analyze the conservation of the miR396 target site.**

**Table S4a. Sequences used to analyze the conservation of the miR396 target site in GRFs.**

|  | cDNA/EST sequence^a^ |
| --- | --- |
| *Arabidopsis thaliana* | At4g37740.1 |
| *Populus trichocarpa* | DT491505.1 |
| *Oryza sativa* | CX116429.1 |
| *Picea glauca* | EX309585.1 |
| *Pinus taeda* | DT624823.1 |

^a^Genebank accession numbers

**Table S4b. Homologs to *Arabidopsis thaliana* *bHLH74* with a miR396 target site.**

|  | Genomic sequence^a^ | cDNA/EST sequence^a^ |
| --- | --- | --- |
| *Arabidopsis lyrata* | ADBK01000024.1 | XM_002892502.1 |
| *Brassica oleracea* | BZ054251.1 | DK459896 |
| *Brassica rapa* | CU984567.1 |  |
| *Brassica napus* | [DU108594.1](http://www.ncbi.nlm.nih.gov/nucleotide/73682702?report=genbank&log$=nuclalign&blast_rank=2&RID=5GS6ERXD012) | EE426459.1 |
| *Raphanus sativus* |  | [EW733962.1](http://www.ncbi.nlm.nih.gov/nucleotide/156173816?report=genbank&log$=nuclalign&blast_rank=13&RID=5GRD6PA9016) |
| *Raphanus raphanistrum* |  | EV538457.1 |
| [*Cleome spinosa*](http://www.ncbi.nlm.nih.gov/Taxonomy/Browser/wwwtax.cgi?name=Cleome+spinosa) |  | GR934263.1 |

^a^Genebank accession numbers
