# Supplementary material for: Functional Specialization of the Plant miR396 Regulatory Network through Distinct MicroRNA–Target Interactions
Source: PLoS Genet. 2012 Jan 5;8(1):e1002419. doi: 10.1371/journal.pgen.1002419 (PMC3252272; doi:10.1371/journal.pgen.1002419)
Supplement: Table S5 — Expression of different miR396 variants in publicly available small RNA sequencing libraries. (DOC) [file pgen.1002419.s012.doc]

**Table S5. Expression of different miR396 variants in publicly available small RNA sequencing libraries.**

| Species | miR396 Variant | Reads | Sequence |
| --- | --- | --- | --- |
| *S. moellendorffii^1^* |  | 39 | UUCCACGGCUUUCUUGAACC |
| *A. thaliana^2^* | a | 11074 | UUCCACAGCUUUCUUGAACUG |
|  | b | 11022 | UUCCACAGCUUUCUUGAACUU |
| *P. trichocarpa^3^* | a,b | 509 | UUCCACAGCUUUCUUGAACUG |
|  | c,d,e | 517 | UUCCACAGCUUUCUUGAACUU |
|  | f,g | 0 | UUCCACGGCUUUCUUGAACU(G/U) |
| *O. sativa^4^* | a,b | 6527 | UUCCACAGCUUUCUUGAACUG |
|  | c | 255872 | UUCCACAGCUUUCUUGAACUU |
|  | e,f | 2529058 | UCCACAGGCUUUCUUGAACUG |
|  | g,h,i | 1466 | UCCACAGGCUUUCUUGAACGG |
| *Brachypodium sp.^5^* | a | 136 | UUCCACAGCUUUCUUGAACUG |
|  | b | 834 | UUCCACAGCUUUCUUGAACUU |
|  | c | 20244 | UCCACAGGCUUUCUUGAACUG |
|  | d | 9 | UCCACAGGCUUUCUUGAACGG |
| *Zea mays^6^* | a,b | 1053 | UUCCACAGCUUUCUUGAACUG |
|  | c,d | 16744 | UCCACAGGCUUUCUUGAACUG |
|  | e,f | 1744 | UUCCACAGCUUUCUUGAACUU |
|  | g,h | - | UCCCACAGCUUUAUUGAACUG |
| *P. contorta^7^* | a | 56 | UUCCACAGCUUUCUUGAACUU |
|  | b | 502 | UUCCACGGCUUUCUUGAACUU |

1. Axtell *et al.*, 2007. The Plant Cell **19**,1750-69.

2. Nakano *et al*., 2006. Nucleic Acids Res. **34**, D731-35 ([*http://mpss.udel.edu/at_sbs/*](http://mpss.udel.edu/at_sbs (Nakano et al., 2006)/))*;* Gustafson *et al.*, 2006. Nucleic Acids Res. **33**, D637-40 ([*http://asrp.cgrb.oregonstate.edu/db /*](http://asrp.cgrb.oregonstate.edu/db /))

3. Klevebring *et al.*, 2009. BMC Genomics **10**, 620.

4. Nakano et al., 2006. Nucleic Acids Res. **34**, D731-35 (<http://mpss.udel.edu/rice_sbs/>)

5. Nakano et al., 2006. Nucleic Acids Res. **34**, D731-35 (http://mpss.udel.edu/brachy_sbs/)

6. Nakano et al., 2006. Nucleic Acids Res. **34**, D731-35 (http://mpss.udel.edu/maize_WGS/)

7. Morin et al., 2008. Genome Res. **18**, 571-584.
