# Supplementary material for: Functional Specialization of the Plant miR396 Regulatory Network through Distinct MicroRNA–Target Interactions
Source: PLoS Genet. 2012 Jan 5;8(1):e1002419. doi: 10.1371/journal.pgen.1002419 (PMC3252272; doi:10.1371/journal.pgen.1002419)
Supplement: Table S6 — Binary plasmids prepared for this study. (DOC) [file pgen.1002419.s013.doc]

**Table S6. Binary plasmids prepared for this study.**

**Reporter and expression vectors.**

| Vector | Construct | Arabidopsis Chromosome: start-end^a^ |
| --- | --- | --- |
| pRER55 | *GRF2-GUS* | [GRF2:*GRF2*, 4:17729683–17725702] - GUS CDS |
|  |  | CGT CAT CGT TCA AGA AAG CCT GTG GAA GTC CAA  R H R S R K P V E V Q |
| pRER56 | *rGRF2-GUS* | [GRF2:*rGRF2*, 4:17729683–17725702] - GUS CDS |
|  |  | CGT CAT CGT TCT AGA AAA CCG GTC GAA GTC CAA  R H R S R K P V E V Q |
| pRER57 | *pGRF2-GUS* | [GRF2:*pGRF2*, 4:17729683–17725702] - GUS CDS |
|  |  | CGT CAT CGT TCA AGA AAG CTG TGG AAa GTC CAA  R H R S R K L W K V Q |
| pJD5 | *bHLH74:wtbHLH74* | 1:3307838–3303948 |
|  |  | TCA CAA TGG AAC AAG[intron]AAA GCT GTG GAG GAA TTT  S Q W N K K A V E E F |
| pJD6 | *bHLH74:rbHLH74* | 1:3307838–3303948 |
|  |  | TCA CAA TGG AAC AAG[intron]AAG GCA GTT GAA GAA TTT  S Q W N K K A V E E F |
| pJD7 | *35S:bHLH74* | 35S:[1: 3305991 – 3304225] |
|  |  | TCA CAA TGG AAC AAG[intron]AAA GCT GTG GAG GAA TTT  S Q W N K K A V E E F |
| pJD8 | *35S:rbHLH74* | 35S:[1: 3305991 – 3304225] |
|  |  | TCA CAA TGG AAC AAG[intron]AAG GCA GTT GAA GAA TTT  S Q W N K K A V E E F |
| pJD22 | *wtbHLH74-GUS* | [bHLH74: *bHLH74*, 1: 3307838 – 3305308] - GUS CDS |
| pJD23 | *rbHLH74-GUS* | [bHLH74:r*bHLH74*, 1: 3307838 – 3305308] - GUS CDS |
| pMM54 | *MIR396b:GUS* | [pmiR396, 5: 13609792 – 13611781] – GUS CDS |

**miR396 and MIM396 expression vectors.**

| Vector | Construct | Expressed sequence^b^ |
| --- | --- | --- |
| pJD38 | *35S:amiR396* | GATCCTAGGAATATATATGTAGCAATTCAAAAAAGGTGTGGATTCACAGGTCGTGATATGATTCAATTAGCTTCCGACTCATTCATCCAAATACCGAGTCGCCAAAATTCAAACTAGACTCGTTAAATGAATGAATGATGCGGTAGACAAATTGGATCATTGATTCTCTTTGATTCCACAGCTTTCTTGAACTGCTCTCTTTTGTATTCCCTGCA |
| pJD65 | *35S:amiR396 7_8insG* | GATCCTAGGAATATATATGTAGCAATTCAAAAAAGGCTGTGGTTCACAGGTCGTGATATGATTCAATTAGCTTCCGACTCATTCATCCAAATACCGAGTCGCCAAAATTCAAACTAGACTCGTTAAATGAATGAATGATGCGGTAGACAAATTGGATCATTGATTCTCTTTGATCCACAG**G**CTTTCTTGAACTGCTCTCTTTTGTATTCCCTGCA |
| pRER7 | *35S:miR396b* | CATGAAGATCCTGGTCATACTTTTCCACAGCTTTCTTGAACTTTCTTTTTCATTTCCATTGTTTTTTTCTTAAACAAAAGTAAGAAGAAAAAAAACTTTAAGATTAAGCATTTTGGAAGCTCAAGAAAGCTGTGGGAAAACATGACAATTCAGGGTTTG |
| pMM18 | *35S:miR396b 7A>G* | CATGAAGATCCTGGTCATACTTTTCCAC**G**GCTTTCTTGAACTTTCTTTTTCATTTCCATTGTTTTTTTCTTAAACAAAAGTAAGAAGAAAAAAAACTTTAAGATTAAGCATTTTGGAAGCTCAAGAAAGCTGTGGGAAAACATGACAATTCAGGGTTTG |
| JD160 | *MIR396b:amiR396* | [pmiR396, 5: 13609792 – 13611781] – *amiR396* |
| JD162 | *MIR396b:amiR396 7_8insG* | [pmiR396, 5: 13609792 – 13611781] – *amiR396 7_8insG* |
| JD57 | *35S:MIM396* | GGTACCGGATCCGCCGTAGCCGGCAGGTCTTCTCCCTCTAGAAATTGTTCAAGAGCTCAGCTGTGGAAAGCTTCGGTTTTTCTCTTTGGAATGTTCAAGAGCGCAGCTGTGGAATTTTTCAATTTTTTTGGTTGGAATGTTCAAGAGCTTAGCTGTGGAATTTTGATGGAAGATCTGCCGTAGCCGGCGTCGAC |

All constructs were cloned in the binary vector pCHF3 (Jarvis, P., Chen, L. J., Li, H., Peto, C. A., Fankhauser, C., and Chory, J. (1998). An Arabidopsis mutant defective in the plastid general protein import apparatus. Science 282, 100103). T‑DNA constructs were introduced into *A. tumefaciens* strain ASE (Fraley, R. T., Rogers, S. G., Horsch, R. B., Eichholtz, D. A., Flick, J. S., Fink, C. L., Hoffmann, N. L., and Sanders, P. R. (1985) The SEV system: a new disarmed Ti plasmid vector system for plant transformation. Biotechnology 3, 629635).

^a^ Higlighted in yellow, nucleotides annealing with miR396. Underlined, mutagenized residues. In red, upstream and downstream sequences. In green, aminoacid changes introduced in *pGRF2.*

^b^ Higlighted in yellow, mature miRNA sequence. In red, mutagenized residues.
