# Supplementary material for: Functional Specialization of the Plant miR396 Regulatory Network through Distinct MicroRNA–Target Interactions
Source: PLoS Genet. 2012 Jan 5;8(1):e1002419. doi: 10.1371/journal.pgen.1002419 (PMC3252272; doi:10.1371/journal.pgen.1002419)
Supplement: Table S7 — Relevant locus identifiers, mutant alleles and RT-qPCR primers. (DOC) [file pgen.1002419.s014.doc]

**Table S7. Relevant locus identifiers, mutant alleles and RT-qPCR primers.**

| Gene | Locus ID | Forward primer | Reverse primer | Mutant allele |
| --- | --- | --- | --- | --- |
| *GRF2* | At4g37740 | CACATCAACAGAGGCCGTCATCG | AACCGGAGATTCCTTGGGTTGTAAG |  |
| *GRF3* | At2g36400 | GTCTTCGCTGGCCACAAGTATT | TGTTGCTGTTGTAGTGGTGGCT |  |
| *GRF4* | At3g52910 | GCCACATTCACCGTGGAAGAAA | CCACTGTTAGCTTCATATGGCCTC |  |
| *GRF5* | At3g13960 | CTCTTCATCATGCTTCCGCTTT | TTGCTAACGGTTGTTGGTGATG |  |
| *PP2A* | At1g13320 | CCTGCGGTAATAACTGCATCT | CTTCACTTAGCTCCACCAAGCA |  |
| *bHLH74* | At1g10120 | GCTACCTGAAGCCGAATCAC (#3) | ATCGAGCATAACCGCTTTTC (#4) | GABY-Kat 720G11 |
|  |  | gtggtaccagaggaATGGGTGGTGA (#1) | ATGGAAGGATGGTAATGAGA (#2) |  |
| *SE* | At2g27100 |  |  | *se-1* (CS3257) |
| *AGO1* | [At1g48410](http://www.arabidopsis.org/servlets/TairObject?id=26776&type=locus) |  |  | *ago1-27* |
| *RDR6* | [At3g49500](http://www.arabidopsis.org/servlets/TairObject?id=126552&type=locus) |  |  | *rdr6-11 (*CS24285) |
| *HYL1* | At1g09700 |  |  | *hyl1-2* (SALK-064863) |
| *DCL1* | [At1g01040](http://www.arabidopsis.org/servlets/TairObject?id=137163&type=locus) |  |  | *dcl1-7* ([CS3089](http://www.arabidopsis.org/servlets/TairObject?type=germplasm&id=1005161319)) |

| Mature miR | RT stem-loop oligo | Forward primer | Reverse primer |
| --- | --- | --- | --- |
| *MIR396b-a* | GTCTCCTCTGGTGCAGGGTCCGAGGTATTCGCACCAGAGGAGACMAGTTC | GGCGGTTCCACAGCTTTCTT | TGGTGCAGGGTCCGAGGTATT |
| *MIR396_7-8insG* | GTCTCCTCTGGTGCAGGGTCCGAGGTATTCGCACCAGAGGAGACMAGTTC | GGCGGTTCCACAGGCTTTCTT | TGGTGCAGGGTCCGAGGTATT |
